# Isolation, Expression Profiling, and Regulation via Host Allelochemicals of 16 Glutathione S-Transferases in the Chinese White Pine Beetle, *Dendroctonus armandi*

**DOI:** 10.3389/fphys.2020.546592

**Published:** 2020-11-12

**Authors:** Haiming Gao, Lulu Dai, Danyang Fu, Yaya Sun, Hui Chen

**Affiliations:** ^1^College of Forestry, Northwest A&F University, Yangling, China; ^2^State Key Laboratory for Conservation and Utilization of Subtropical Agro-Bioresources, Guangdong Key Laboratory for Innovative Development and Utilization of Forest Plant Germplasm, College of Forestry and Landscape Architecture, South China Agricultural University, Guangzhou, China; ^3^College of Forestry, Nanjing Forestry University, Nanjing, China

**Keywords:** transcript levels, terpenoids, host phloem, glutathione S-transferases, Chinese white pine beetle

## Abstract

The Chinese white pine beetle (*Dendroctonus armandi*) is undoubtedly one of the most important pests causing ecological damage in the Qinling Mountains. When bark beetles invade conifers, they must overcome host tree defenses, including primary resistance and induced resistance responses. Moreover, this induced resistance occurs following herbivory by bark beetles. Bark beetles have a corresponding defense mechanism for degrading toxic compounds, and glutathione S-transferases (GSTs) can catalyze the binding of endogenous substances that reduce glutathione (GSH) to various harmful electrophilic substrates, increasing their solubility and facilitating their excretion from cells. In this experiment, we successfully obtained sixteen full-length sequences of *D. armandi*, which belonged to four GST categories (delta, epsilon, sigma, and theta). The transcript levels of sixteen GSTs in *D. armandi* were compared at four developmental stages (larvae, pupae, teneral adults, and adults), three different tissues (antennae, gut, and reproductive organs), and under various levels of terpenoid stimuli during feeding on phloem tissue to evaluate the various relevant modes of action. This study aids in the understanding of the interaction between monoterpenes and beetles, and beetles’ detoxification through GSTs.

## Introduction

Bark beetles in Scolytinae, including genera *Dendroctonus*, *Ips*, and *Scolytus*, are the most important pests affecting forest health north of the Equator (Schelhaas et al., 2003; Raffa et al., 2008; Bentz et al., 2010; Marini et al., 2017). *Dendroctonus* species are particularly harmful to conifers (Haack and Frank, 1988). Although many species of bark beetles multiply in injured or dying trees, some also invade healthy hosts and reach high population densities during outbreaks (Rudinsky, 1966; Schenck and Benjamin, 1969; Furniss et al., 1979; Wood, 1982; Wright et al., 1984; Miller et al., 1989; Klepzig et al., 1996; Schroeder, 1999; Wallin and Raffa, 2001).

The Chinese white pine beetle, *Dendroctonus armandi* (Coleoptera: Curculionidae; Scolytinae) is one of the most harmful pests in the Qinling Mountain ecosystem (Yin et al., 1984; Chen and Yuan, 2000). Various native insects, such as *D. armandi*, can undergo population increases that reach epidemic levels, leading to widespread tree death in the forest ecosystem south of the Qinling Mountains. *D. armandi* has mainly damaged healthy Chinese white pine (*Pinus armandi*) over the past 30 years or more (Chen and Tang, 2007). The resin contains volatile monoterpenes and sesquiterpenes include α-pinene, β-pinene, β-caryophyllene, and β-myrcene, which, produced by the damaged tree, are harmful for beetles (Reid and Purcell, 2011; Dai et al., 2015). Moreover, the terpenoid substances can also be used by beetles for the colonization and selection of hosts (Delorme and Lieutier, 1990; Raffa and Smalley, 1995; Klepzig et al., 1996). Furthermore, monoterpenes are indispensable substances in pheromone precursor synthesis for beetles (Lindström et al., 1989; Seybold, 1993; Byers, 1995) or serve as the main attractant of other herbivores and even fungal symbionts (DiGuistini et al., 2011).

The beetles must first survive exposure to the toxic substances and then convert these terpenoids into other substances. Bark beetles have a corresponding defense mechanism for degrading toxic compounds, composed primarily of cytochromes P450 (CYP), esterases, and glutathione S-transferases (GSTs), which are the three major multi-gene family enzymes involved in this defense (López et al., 2013).

GSTs (EC 2.5.1.18) are a class of supergene families that exist as dimers and are ubiquitous in nature, widely found in animals (insects), plants (beans), microbes (fungi, bacteria), and the human body (Enayati et al., 2005; Malik et al., 2016; Skopelitou et al., 2017; Alqarni et al., 2018). GSTs are enzymes that can catalyze the binding of endogenous substances that reduce glutathione (GSH) to various harmful electrophilic substrates, increasing their solubility and facilitating their excretion from cells (Hayes et al., 2005).

Various insect life stages are constantly exposed to exogenous and endogenous toxic substances such as pesticides and plant defense allelochemicals, which are needed to degrade toxins via GSTs in insects (Ketterman et al., 2011). The cytoplasmic GSTs in insects are mainly divided into six categories: delta, epsilon, omega, sigma, theta, and zeta. And GSTs in epsilon and delta are insect-specific which have the largest number of Coleoptera species like *Tribolium castaneum* (Shi et al., 2012). Further study also shows that delta and epsilon class GSTs are specifically involved in insecticide resistance in *Meligethes aeneus* to the most commonly used pyrethroids (Erban and Stara, 2014).

GSTs function mainly in the cell, inform the active electrophilic group attack, through the catalytic reduction of GSH, and participate in the detoxification process along with other detoxification enzymes (Sheehan et al., 2001). The GST enzymes activity and GSH content have been studied for a long time, which were considered as an important element in the insecticide resistance of beetles (Reidy et al., 1990). But their functions in the detoxification of the host chemical defense were concerning. Previous study has reported that GSTs have been cloned from the spruce budworm *Choristoneura fumiferana*, which mainly feeds on conifer tissues (Feng et al., 2001). Moreover, studies on the GST gene of mountain pine beetles *Dendroctonus ponderosae* have included the functional annotation of the expressed sequence tags and the resulting genome (Keeling et al., 2012, 2013). Robert et al. (2013) performed a transcriptome sequencing analysis (RNA-seq) on males and females after starvation or feeding for 24 h and showed a significant increase in some GST transcripts after feeding on lodgepole pine *Pinus contorta* tree tissue. And the insect physiology conditions like life-stage and sex should been considered in studying GST enzymes activity under toxic stress (Wilczek et al., 2003).

In this study, we isolated sixteen GST genes of *D. armandi*, and detected their expressions at different life history stages, and in different organs and tissues. The main goal of this study was to determine the response of GSTs in Chinese white pine beetles when exposed to terpenoids or feeding on host phloem.

## Materials and Methods

### Collection of Insect Samples

Samples were collected from infested *P. armandi* at the southern slope of the Qinling Mountain, Shaanxi Province, China (33° 18′-33° 28′ N, 108° 21′-108° 29′ E). At the end of April 2018, 1.0–1.5 m logs were cut from infested trees, transported to the laboratory, and placed in a pot with moist sand. The outer layer was covered with gauze. Hundreds of larvae, pupae, and adults were collected from the logs. The adults were divided into three sub-stages: teneral adults (brightly colored body), emerged adults, and feeding adults (those who invaded a new host) according to their different behaviors (Dai et al., 2014). Only the latter two sub-stages: emerged and feeding adults were separated by the sex according to the back view of the abdomen (Lyon, 1958). Then, beetles were placed in a plastic container filled with wet paper and transported at 4°C.

### Isolation of GST Genes

Total RNA was isolated from 9 larvae, 6 pupae, 3 teneral adults (indicated by a brightly colored body), 6 emerged adults (1:1 ratio), and 6 feeding adults (1:1 ratio) (those feeding on new conifers) following the manufacturer’s instructions of the UNIQ-10 Column TRIzol Total RNA Isolation Kit (Sangon Biotech, Shanghai, China). The obtained RNA was checked for integrity using 1% agarose gel electrophoresis, and its quality was examined with a NanoDrop 2000 (Thermo Scientific, Pittsburgh, Pennsylvania, United States). The cDNA template strand was synthesized with the FastKing RT Kit (with gDNase) (Tiangen, Beijing, China) according to the instruction manual.

Specific primers (Supplementary Table S1A) were designed for each gene of interest using the Primer Premier 5.0 software based on an existing transcriptome database (Dai et al., 2015). PCR amplification of the cDNA was performed with a C1000 thermocycler (Bio-Rad Laboratories, Hercules, CA, United States). The reaction system was 20 μL in volume, including 1 μL of cDNA, 0.5 μL of each primer (10 μM), and 10 μL of 2 × *EasyTaq* PCR SuperMix (TransGen Biotech, Beijing, China). The reaction conditions were 30 s at 94°C, 30 cycles of 30 s at 94°C, 30 s at 55–62°C, 1 min at 72°C, and a last extension step of 10 min at 72°C.

The PCR products were aligned with a 2K Plus DNA Marker (TransGen Biotech) in 1% agarose gel electrophoresis, where the agarose gel was stained with 1 μL GelStain 10,000× (TransGen Biotech). The resulting PCR product was further purified using a Gel Purification Kit (Omega, GA, United States), and the resulting DNA was ligated into the pMD^TM^ 18-T Vector (TaKaRa Bio Inc., Dalian, China) and then transformed into DH5α chemically competent cells of *Escherichia coli* (TransGen Biotech). After the bacterial solution was applied to the plate and cultured overnight in an incubator, white colonies were transferred to Amp/X-Gal/IPTG/LB plates, and then three replicates per plate were sent to Sangon Biotech (Shanghai, China) for sequencing. Partial fragments of the sequence were returned by sequencing, and the partial sequences were compared and analyzed with the National Center Biotechnology Information database^1^ by manual editing with DNAMAN (v.7.0).

The partial sequences identified above were used for the primer design to amplify the complete sequences of the GST genes with a SMARTer RACE cDNA Amplification Kit (Clontech Laboratories Inc., Mountain, CA, United States). The amplicons were purified, cloned, and sequenced as described previously. To avoid chimera sequences, specific primers (Supplementary Table S1C) were designed based on the obtained complete sequences to amplify the complete DNA of each GST gene. Amplification reactions were carried out in 20 μL volumes containing: 1 μL cDNA, 0.25 μM of each primer, and 10 μL 2 × *EasyTaq* PCR SuperMix (TransGen Biotech, Beijing, China). The PCR reaction was performed as follows: 94°C for 5 min; 30 cycles of 94°C for 30 s, Tm of each pair of primers for 30 s and 72°C for 1 min; and a final extension for 10 min at 72°C. PCR products were visualized on 1% agarose gels, purified, cloned, and sequenced for both strands as described above.

The full-length sequence of the GST genes were translated into an amino acid using the Open Reading Frame Finder^2^, searched against the NCBI database using BLASTp (Altschul et al., 1990).

### Full-Length Sequence Analysis of GST Genes

The DNA sequences and the translated amino acid sequences were searched against the NCBI nr database using blastx and analyzed in the GenBank database on the NCBI website^1^. The amino acid sequence was BLAST-searched with the existing insect GST sequences in the GenBank database, and the GST gene sequences that were fully verified were uploaded to GenBank (accession numbers: MK796851-MK796867).

A total of 44 GST sequences of other insects were selected for the phylogenetic analysis according to the BLASTp searching used by deduced amino acid sequences of the obtained *DaGSTs*. The phylogenetic tree of *DaGSTs* was constructed with the maximum likelihood method using the Mega 5.0 (Tamura et al., 2013) software. The Whelan and Goldman (WAG) model was supported by best fitting model testing in Mega 5.0 (−ln *L* = 5008.27) with a gamma parameter value *G* = 2.70. The bootstrap value was calculated after 500 pseudoreplicates to estimate the support for each node.

The GST amino acid sequences were subjected to multiple sequence alignment analysis with CLUSTALX v.2.0 (Thompson et al., 1997), using the default software settings. The secondary structure templates of the GST proteins were obtained from the Brookhaven Protein Data Bank website^3^, and crystal structures (Delta and Epsilon: 4YH2; Sigma: 5H5L; Theta: 5ZFG) were used for alignment and analysis. The results of the alignment were visualized with ESPript (Robert and Gouet, 2014), and the secondary structure was assigned to the corresponding sequence.

The molecular weight (kDa) and isoelectric point of the obtained GST gene sequences were analyzed using ProtParam (Gasteiger et al., 2005). The resulting GST amino acid sequence was then subcellularly localized using the TargetP^4^ (Emanuelsson et al., 2000) website, and its function was predicated with the default parameters.

### Different Development Stages and Treatments

#### Development Stages

Samples of five developmental stages of *D*. *armandi* were collected: larvae, pupae, and adult which divided into three sub-stages: teneral adults (brightly colored body), emerged adults, and feeding adults (those who invaded a new host). Only emerged and feeding adults were separated by sex.

#### Feeding Treatments

A total of 180 emerged adults (90 each ♀/♂) were selected and separated according to sex with 30 individuals of each sex placed into one group. One group was placed in Petri dishes (90 × 15 mm) containing new filter paper and stored in a dark environment for 2 h as a control. The other two beetle groups were placed in 2 mL centrifuge tubes contains 2 × 2 cm sections of fresh host phloem one by one, and placed in a dark environment for 12 and 24 h. After feeding treatment, viability was observed under a dissecting microscope. As no beetles died after feeding treatment, for every group, 12 beetles of each sex were randomly selected for real-time qPCR detection.

#### Terpenoid Treatment

Thirty beetles of different stages: larvae, pupae, teneral, and emerged adults (only emerged adults divided by sex) were placed in a 90 mL Petri dish (90 × 15 mm) containing moist filter paper, respectively. A dry filter paper was placed inside the top of the dish. The terpenoids used in this experiment were identical to those in the fumigant toxicity tests previously performed by the team (Dai et al., 2014). The five terpenoids used were as follows: (−)-α-pinene (98%), (−)-β-pinene (99%), (+)-3-carene (90%), (±)-limonene (95%), and turpentine oil (Aladdin Industrial Corporation, Shanghai, China). The terpenoids were added to the top filter paper at a concentration of 1,000 ppm, and the Petri dish was immediately sealed with Parafilm. Each terpenoid treated three Petri dishes of each stage. The treated dishes were placed in an incubator in a dark environment at room temperature for 8 h and 24 h. Insect viability in each dish was examined under a dissecting microscope after fumigation with the terpenoids. With 20% mortality, 12 living beetles were selected for real-time qPCR detection. Untreated beetles of each stage were used as controls.

Furthermore, other groups with emerged adults were treated with terpenoids at 1,000 ppm for 8 h, performed as above. And antennal, gut, and reproductive tissues of emerged adult beetles were dissected, and used for real-time qPCR detection.

### Real-Time qPCR

RNA extraction and first-strand cDNA synthesis were performed according to the cloning of GST genes method. Specific primers for each sequence were designed using Primer Premier 5.0 based on the full-length gene sequences obtained. The RT-PCR system had a total reaction volume of 20 μL, including 0.4 μL per primer (10 μmoL⋅L^–1^), 1 μg of cDNA template, and 10 μL of Roche SYBR Green mix (Roche Diagnostics Mannheim, Germany). All reactions were performed with a CFX96^TM^ Real-Time PCR Detection System (Bio-Rad). The reaction conditions were as follows: 95°C for 30 s, 95°C for 5 s for 40 cycles, Tm (melting temperature) for each pair of primers (Supplementary Table S1D) for 15 s, and 72°C for 20 s. Three biological replicates plus three technical replicates were used.

The amplification efficiency and effectiveness of the RT-qPCR for different genes were analyzed by linear regression between the dilutions of the different concentration gradients (1.0, 10^–1^, 10^–2^, 10^–3^, 10^–4^, 10^–5^, 10^–6^, 10^–7^, 10^–8^) of the cDNA for quantification. The processed cDNA template was diluted into a concentration gradient, and 5 μL of each RT-qPCR template from the previous concentration gradient dilution was added. PCR was performed three times for each gene, and its efficiency was estimated as follows: efficiency = (10^–1/slope^−1) × 100, where the efficiency value was 100% ± 10% (Supplementary Table S1D). The estimated PCR verification was verified by *R*^2^-values, which were *>* 0.90. In addition, it should be noted that amplification primers specificity was to be determined by melting curves.

The transcriptional expression levels of *D. armandi* GSTs were normalized to the transcript levels of β-*actin* (KJ507199) and *CYP4G55* (KR012821), as these two genes have stable expression levels in different stages, tissues, and feeding or terpenoids treatment (Dai et al., 2019). By calculating the geometric mean of these two genes, the accuracy of the relative expression level results can be improved. The 2^–ΔΔ*Ct*^ method was used to determine the relative expression of *D. armandi* GSTs (Schmittgen and Livak, 2008). To evaluate the significant difference between different treatments, all of the 2^–ΔΔ*Ct*^ values were log_2_ transformed.

One-way ANOVA analyzed the transcriptional expression levels of *D. armandi* GSTs in different development stages and different time points of feeding/fumigation treatments of each stage, sex, and stimulus with a Tukey test. And the difference between the sexes of adult beetles was performed with independent-samples *T*-tests. The expression profiles of the GSTs in different tissues of each sex were analyzed with two-way ANOVA including interaction. Statistical analysis was performed using SPSS 18.0 (IBM SPSS Statistics, Chicago, IL, United States), and the results were plotted with GraphPad Prism 6.0 (GraphPad Software, CA, United States).

## Results

### Sequence Analysis of Full-Length *D. armandi* GST Genes

**FIGURE 1:**
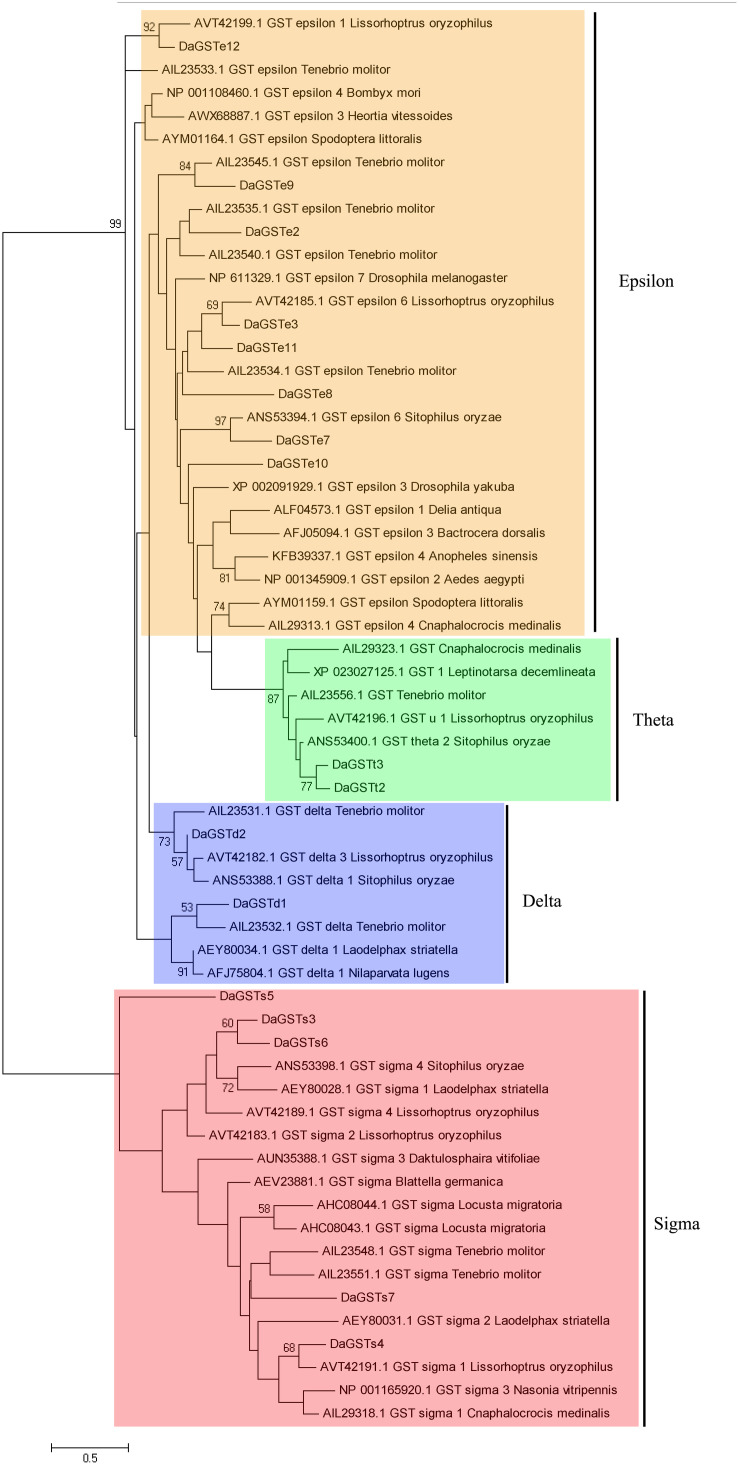
Phylogenetic analysis of sequences of GST genes from *Dendroctonus armandi* was performed using the amino acid substitution model WAG+ G (–ln*L* = 5008.27, G = 2.7011). The matched genes are listed by the accession numbers of the corresponding sequence in GenBank. Bootstrap values after 500 pseudoreplicates are shown as nodes. The evolutionary branch is divided into four parts: epsilon is orange, theta is green, delta is blue, and sigma is red.

A total of 16 full-length GST codon sequences were obtained from *D. armandi* which belonged to the delta, epsilon, sigma, and theta families. They were named as *DaGSTs* according to their homology with the GSTs of other insects (Table 1 and Figure 1). The open reading frame (ORF) of the GST gene ranges from 603 bp (*DaGSTs6*) to 696 bp (*DaGSTt2* and *DaGSTt3*), and its coding region encodes a polypeptide consisting of approximately 200 amino acids. The ProtParam software predicted the molecular weight to be approximately 22.984 kDa (*DaGSTs6*) to 26.792 kDa (*DaGSTt2*), while the theoretical isoelectric point ranged from 4.41 (*DaGSTe9*) to 9.05 (*DaGSTs5*). According to the location of the cells, the GSTs we cloned belonged to the cytosolic class (Table 1).

**TABLE 1:** Physicochemical properties and cellular localization of putative *Dendroctonus armandi* GST proteins.

GST name	Accession number	ORF size (aa\bp)^a^	MW (kDa)^a^	IP^a^	Signal peptide prediction^b^
*DaGSTd1*	MK796851	221\666	24.415	4.95	mTP 0.303, SP 0.141, other 0.447
*DaGSTd2*	MK796852	218\657	24.531	4.98	mTP 0.100, SP 0.160, other 0.754
*DaGSTe2*	MK796853	217\654	24.492	6.59	mTP 0.118, SP 0.469, other 0.254
*DaGSTe3*	MK796854	218\657	24.656	7.80	mTP 0.265, SP 0.142, other 0.496
*DaGSTe7*	MK796855	221\666	24.932	7.05	mTP 0.093, SP 0.406, other 0.482
*DaGSTe8*	MK796856	217\654	24.875	5.74	mTP 0.057, SP 0.650, other 0.231
*DaGSTe9*	MK796857	219\660	24.776	4.41	mTP 0.082, SP 0.452, other 0.396
*DaGSTe10*	MK796858	221\666	25.500	5.35	mTP 0.125, SP 0.198, other 0.752
*DaGSTe11*	MK796859	218\657	23.612	6.83	mTP 0.071, SP 0.525, other 0.523
*DaGSTs3*	MK796861	207\624	23.273	8.58	mTP 0.276, SP 0.085, other 0.632
*DaGSTs4*	MK796862	205\618	23.297	5.97	mTP 0.057, SP 0.400, other 0.639
*DaGSTs5*	MK796863	211\636	24.003	9.05	mTP 0.156, SP 0.070, other 0.086
*DaGSTs6*	MK796864	200\603	22.984	8.45	mTP 0.258, SP 0.092, other 0.643
*DaGSTs7*	MK796865	203\612	23.316	7.79	mTP 0.096, SP 0.116, other 0.812
*DaGSTt2*	MK796866	231\696	26.792	5.36	mTP 0.092, SP 0.343, other 0.443
*DaGSTt3*	MK796867	231\696	26.720	5.88	mTP 0.056, SP 0.442, other 0.533

*^*a*^Predicted by the ProtParam program. ^*b*^Predicted by TargetP 1.1; SP denotes signal peptide; mTP denotes mitochondrial targeting peptide.*

**FIGURE 2:**
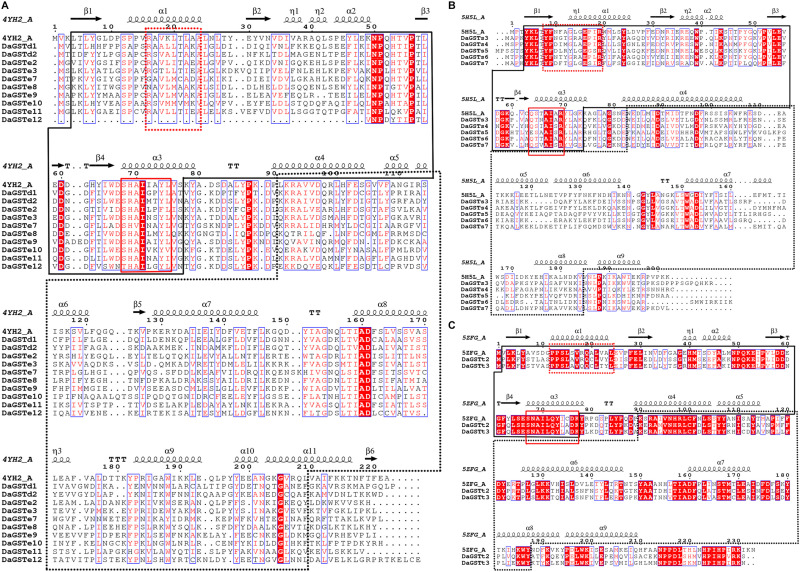
Multiple sequence alignment and secondary structure element assignment of GSTs from *Dendroctonus armandi*. The alignment included *DaGST*s (delta, epsilon, sigma, and theta) and the predicted *Drosophila melanogaster* glutathione S-transferase e6 protein sequence (PBDID: 4YH2, Chain: A) **(A)**, prostaglandin synthase D of *Nilaparvata lugens* glutathione S-transferase s2 protein sequence (PBDID: 5H5L, Chain: A) **(B)** and prostaglandin synthase D of silkworm (*Bombyx mori*) glutathione S-transferase (PBDID: 5ZFG, Chain: A) **(C)**. Alpha helices are marked as alpha or beta based on automatic assignment according to the template for the three (glutathione S-transferase e6, glutathione S-transferase s2, and glutathione S-transferase) protein structures in ESPript. The black solid line box region is the GST N-terminal domain; the dashed line box region is the GST C-terminal domain; the red solid line box region is the characteristic GST motif; the red dashed line box region is the characteristic motif of different families of GSTs.

The *DaGSTs* gene of *D. armandi* and its GSTs have high homology with those of other insects according to multiple sequence alignment analysis. Multiple comparisons of *DaGSTs* with other insect GST amino acid sequences also showed that the N-terminal domain and the C-terminal domain in the secondary structure are β −α −β −α −β −β −α and 5-6 α-helices, respectively (Figure 2). The *DaGSTs* in *D. armandi* had high similarity with four types of amino acids of other insects (Table 2). The maximum likelihood phylogenetic tree showed that most of GSTs from *D. armandi* were gathered with GSTs from beetles in Curculionidae like *Lissorhoptrus oryxophilus* and *Sitophilus oryzae* (Figure 1). But some GSTs in the sigma family from the Chinese white pine beetle had low homology with known GSTs in the NCBI databases (Figure 1). Based on comparison analysis, *DaGST*s have greater than 34% identity with GSTs in other insects (Table 2), which also agrees with the viewpoint of Chelvanayagam’s proposed GST classification standard (Chelvanayagam et al., 2001).

**TABLE 2:** Putative amino acid identity of GST cDNA isolated from *Dendroctonus armandi* and GST sequences from other species.

Genes	BLAST matches in GenBank			Identity % in full length (BLASTp)^*a*^
	Species	GST name	Accession number	
*DaGSTd1*	*Dendroctonus ponderosae*	GST	XP_019764527.1	95
	*Anoplophora glabripennis*	GST	XP_018568551.1	76
	*Tenebrio molitor*	GST delta	AIL23532.1	74
*DaGSTd2*	*Lissorhoptru soryzophilus*	GST delta 3	AVT42182.1	79
	*Oryctes borbonicus*	GST	KRT80357.1	71
	*Blattella germanica*	GST	CAO85744.1	68
*DaGSTe2*	*Tenebrio molitor*	GST epsilon	AIL23540.1	46
	*Fopius arisanus*	GST	XP_011314548.1	46
	*Sitophilus oryzae*	GST epsilon 2	ANS53390.1	46
*DaGSTe3*	*Lissorhoptrus oryzophilus*	GST epsilon 2	AVT42186.1	51
	*Anoplophora glabripennis*	GST 1	XP_018564051.1	48
	*Drosophila arizonae*	GST	XP_017865872.1	44
*DaGSTe7*	*Sitophilus oryzae*	GST epsilon 6	ANS53394.1	61
	*Lissorhoptrus oryzophilus*	GST epsilon 5	AVT42201.1	52
	*Sitophilus oryzae*	GST epsilon 3	ANS53391.1	48
*DaGSTe8*	*Danaus plexippus plexippus*	GST	OWR45830.1	41
	*Bombyx mori*	GST epsilon 1	NP_001037197.1	40
	*Onthophagus taurus*	GST	XP_022905048.1	39
*DaGSTe9*	*Tenebrio molitor*	GST epsilon	AIL23539.1	44
	*Agrilus planipennis*	GST	XP_018333399.1	44
	*Tribolium castaneum*	GST 1	XP_008200938.1	41
*DaGSTe10*	*Drosophila melanogaster*	GST epsilon 2	NP_611324.1	40
	*Aethina tumida*	GST	XP_019878054.1	40
	*Drosophila erecta*	GST 1	XP_026836720.1	38
*DaGSTe11*	*Dendroctonus ponderosae*	GST	XP_019755792.1	44
	*Drosophila rhopaloa*	GST	XP_016984875.1	42
	*Plutella xylostella*	GST	AHW45903.1	42
*DaGSTe12*	*Lissorhoptrus oryzophilus*	GST epsilon 1	AVT42199.1	84
	*Tenebrio molitor*	GST epsilon	AIL23533.1	69
	*Helicoverpa armigera*	GST	XP_021183202.1	43
*DaGSTs3*	*Sitophilus oryzae*	GST sigma 4	ANS53398.1	53
	*Lissorhoptrus oryzophilus*	GST sigma 4	AVT42189.1	52
	*Solenopsis invicta*	GST	XP_025988454.1	50
*DaGSTs4*	*Anoplophora glabripennis*	GST	XP_018560948.1	61
	*Locusta migratoria*	GST sigma 1	AEB91973.1	50
	*Rhopalosiphum maidis*	GST	XP_026820793.1	50
*DaGSTs5*	*Halyomorpha halys*	GST	XP_014285065.1	38
	*Aphidius gifuensis*	GST	AWS20692.1	34
	*Lissorhoptrus oryzophilus*	GST sigma 2	AVT42183.1	34
*DaGSTs6*	*Zootermopsis nevadensis*	GST	XP_021935879.1	46
	*Sitophilus oryzae*	GST sigma 3	ANS53397.1	46
	*Orussus abietinus*	GST	XP_012276764.1	46
*DaGSTs7*	*Laodelphax striatella*	GST sigma 2	AEY80031.1	49
	*Cnaphalocrocis medinalis*	GST sigma 1	AIL29318.1	50
	*Tenebrio molitor*	GST sigma	AIL23551.1	49
*DaGSTt2*	*Sitophilus oryzae*	GST theta 2	ANS53400.1	69
	*Cnaphalocrocis medinalis*	GST	AIL29323.1	58
	*Apis mellifera*	GST 1	XP_026296300.1	56
*DaGSTt3*	*Onthophagus taurus*	GST 1	XP_022905921.1	58
	*Papilio polytes*	GST 1	NP_001298693.1	57
	*Heortia vitessoides*	GST	AWX68896.1	57

*^*a*^Predicted by BLASTp (https://www.ncbi.nlm.nih.gov).*

### RT-qPCR

#### Different Developmental Stages

**FIGURE 3:**
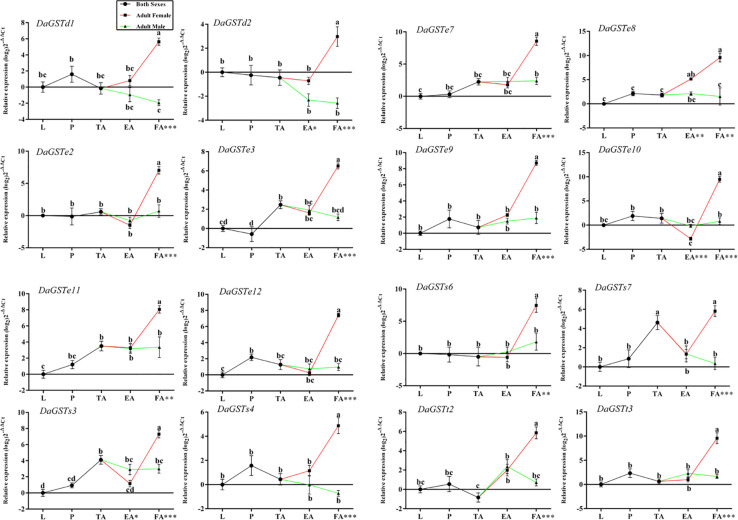
Quantitative expression of GST genes (mean ± SE, *n* = 3) in different developmental stages of *Dendroctonus armandi*. L, larvae; P, pupae; TA, teneral adults; EA, emerged adults; FA, feeding adults, females and males of the two latter stages of *Dendroctonus armandi*. GST expression was normalized to the transcription levels geometric mean of β-*actin* and *CYP4G55*. Different letters indicate significant differences from the different development stages (*P* < 0.05) by Duncan’s test. ^∗^Significant differences between sexes of the same stage as measured by independent Student’s *t*-tests (^∗^*P* < 0.05, ^∗∗^*P* < 0.01, ^∗∗∗^*P* < 0.001). The 2^− ΔΔCt^ and SE values were log_2_ transformed for plotting (*DaGST*s expression is upregulated if the −^ΔΔ^Ct has a positive value, and downregulated for negative values). Statistically significant differences in gene expression are shown in Supplementary Table S2.

Significant differences in the GST expression levels among the different developmental stages were identified with one-way ANOVA (Supplementary Table S2). The transcription levels of most of the GST genes had significant differences among different stages (Figure 3). Between sexes, only *DaGSTd2*, *DaGSTe8*, *DaGSTe10*, and *DaGSTs3* had significant differences in emerged adults (Figure 3). Moreover, in feeding adults, the expression levels of all GSTs significantly differed between sexes (Figure 3).

In comparison with the expression levels in the larval stage, many *DaGSTs* were upregulated in the pupal stage, and most of them were upregulated in teneral adults (Figure 3). All *DaGST*s were upregulated in feeding females, but *DaGSTd1*, *DaGSTd2*, and *DaGSTs4*, were downregulated in males (Figure 3).

#### Feeding Treatment

**FIGURE 4:**
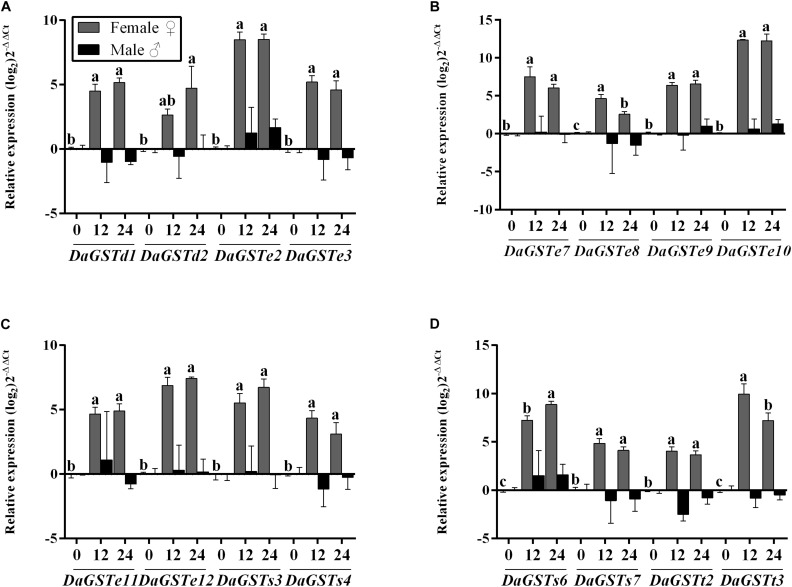
Quantitative transcript levels of GST genes (mean ± SE, *n* = 3) in feeding adults (12 and 24 h) of *Dendroctonus armandi*. GST expression was normalized to the transcription level geometric mean of β-*actin* and *CYP4G55*. Different letters indicate significant differences at *P* < 0.05 (with Tukey test, lowercase for females and uppercase for males, no letter means no significant difference among times). Statistically significant differences in gene expression are shown in Supplementary Table S3.

Significant differences were found in the expression levels of all GST genes after feeding on host phloem for 12 and 24 h in females, but only *DaGSTt2* had obvious downregulation in males (Figure 4 and Supplementary Table S3).

Females over expressed all GSTs in comparison to starvation adults after feeding on host phloem. But the transcription levels of *DaGSTe8* and *DaGSTt3* were higher at 12 h after feeding on host phloem than 24 h (Figure 4 and Supplementary Table S3).

#### Terpenoid Treatments

One-way ANOVA of 16 GST gene expression levels were performed after terpenoid treatment for 8 and 24 h in different stages of *Dendroctonus armandi* with a Tukey test (Supplementary Tables S4, S5).

**FIGURE 5:**
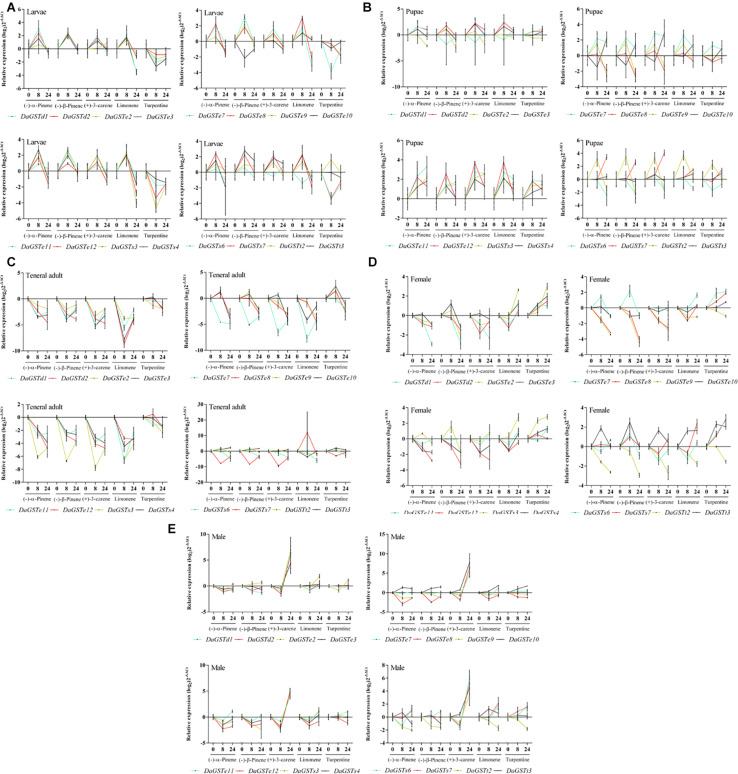
Quantitative expression of GST genes (mean ± SE, *n* = 3) in different life stages (**A:** larvae; **B:** pupae; **C:** teneral adult; **D:** emerged female adults; **E:** emerged male adults) of *Dendroctonus armandi* after stimulation with five terpenoids at exposure times of 0, 8, and 24 h. GST expression was normalized to the transcription level geometric mean of β-*actin* and *CYP4G55*. The 2^– ΔΔCt^ and SE values were log_2_ transformed for plotting. Statistically significant differences in gene expression are shown in Supplementary Tables S4, S5.

In the larval stage, a few *DaGSTs* were significantly upregulated after 8 h of exposure with (−)-α-pinene, (−)-β-pinene, and (±)-limonene, while *DaGSTe12*, *DaGSTs3*, and *DaGSTs6* were significantly downregulated after exposure with turpentine (Figure 5A and Supplementary Table S5).

For most *DaGSTs*, the transcription level in pupae was insensitive to all five stimuli. *DaGSTe7*, *DaGSTe12*, and *DaGSTs3* were significantly upregulated at 8 h after exposure with (+)-3-carene or (±)-limonene. *DaGSTt2* was significantly upregulated at 8 h after exposure with (−)-α-pinene, (−)-β-pinene, (+)-3-carene, and (±)-limonene (Figure 5B and Supplementary Table S5).

After exposure with (−)-α-pinene, (−)-β-pinene, (+)-3-carene, (±)-limonene, the transcription levels of most GSTs were significantly downregulated in teneral adults at all exposure times. But the transcription levels in a few of the GSTs changed after exposure with turpentine (Figure 5C and Supplementary Table S5).

The transcription levels of most GSTs in females were significantly changed after treatment with terpenoids. But the variation trends of different genes were diversiform with different stimuli. (−)-α-pinene and (−)-β-pinene treatment significantly downregulated *DaGSTd1*, *DaGSTe8*, *DaGSTe9, DaGSTe12*, *DaGSTs4*, and *DaGSTt2*. But (±)-limonene and turpentine treatment significantly upregulated *DaGSTe2, DaGSTe3, DaGSTe7, DaGSTs3, DaGSTs7*, and *DaGSTt3*. The transcription levels of *DaGSTe9* and *DaGSTt2* were significantly downregulated after treatment with (±)-limonene and turpentine (Figure 5D and Supplementary Table S5).

The response of GSTs in males to terpenoids treatment was unlike females. After exposure with (+)-3-carene almost every GST was significantly overexpressed in males at 24 h. But only (−)-α-pinene, (−)-β-pinene, (±)-limonene, and turpentine caused significant downregulation in some GSTs like *DaGSTe2*, *DaGSTe8, DaGSTe11*, and *DaGSTt2* after an exposure of 8 h (Figure 5E and Supplementary Table S5).

#### Different Tissues Without Any Treatment

**FIGURE 6:**
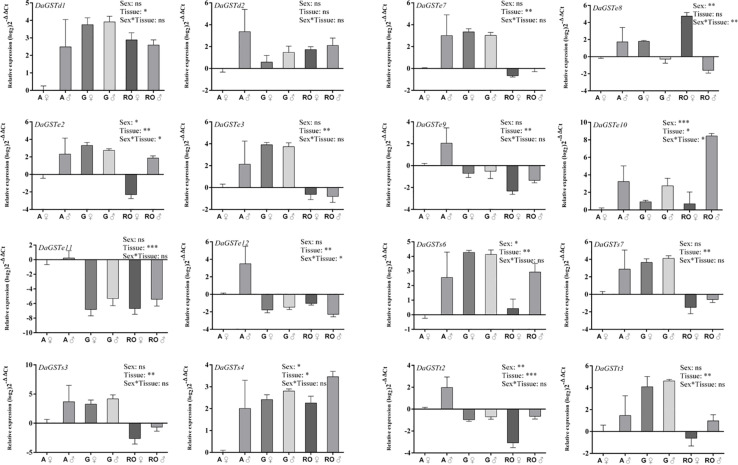
Quantitative expression of GST genes in different tissues of different sexes in *Dendroctonus armandi*. Antennae (A), gut (G), and reproductive organs (RO). GST expression was normalized to the transcription level geometric mean of β-*actin* and *CYP4G55*. Statistically significant differences in gene expression between sexes, tissues and sex*tissue are indicated with asterisks (^∗^*P* < 0.05, ^∗∗^*P* < 0.01, ^∗∗∗^*P* < 0.001). The 2^– ΔΔCt^ and SE values were log_2_ transformed for plotting. Statistically significant differences in gene expression are shown in Supplementary Table S6.

The significant differences between sexes were found in the expression level of six GSTs (*DaGSTe2*, *DaGSTe8*, *DaGSTe10*, *DaGSTs4*, *DaGSTs6*, and *DaGSTt2*), but significant differences were found in 14 GSTs among different tissues. The significant differences were only found in the expression level of *DaGSTe2*, *DaGSTe8*, *DaGSTe10*, and *DaGSTe12* for sex^∗^tissues interaction (Figure 6 and Supplementary Table S6).

#### Different Tissues After Terpenoid Treatments

After treatment with (−)-α-pinene, more than half of the GSTs were significantly overexpressed in the gut of females and reproduction organ of males (Supplementary Table S7). In contrast, most of them were significantly downregulated in the gut of males and reproduction organ of females after treatment with (+)-3-carene. The treatment with (−)-β-pinene only caused two GST genes (*DaGSTe8* and *DaGSTe11*) to be overexpressed in the antennae of females, and two genes (*DaGSTd2* and *DaGSTs7*) to be downregulated in the gut of males. The (±)-limonene and turpentine induced a few GSTs of epsilon in the antennae, gut, or reproduction organ of females (Table 3).

**TABLE 3:** Significant difference of GST genes express trend (mean ± SE, *n* = 3) in different adult tissues [antennae (A), gut (G), and reproductive organs (RO)] of *Dendroctonus armandi* after terpenoids treatment for 8 h.

Tissue	(–)-α-pinene		(–)-β-pinene		(+)-3-carene		(±)-limonene		Turpentine	
	↑	↓	↑	↓	↑	↓	↑	↓	↑	↓
A♀	*e8, e12*	None	*e8, e11*	None	None	*e10, e11*	None	None	*e8, e9, e10, e12*	None
A♂	None	None	None	None	None	None	None	None	None	None
G♀	*d1, e3, e8, e9, e10, e11, e12, s3, s4, t2*	None	None	None	None	None	*e8, e12*	None	*e10, t2*	None
G♂	*e10*	None	None	*d2, s7*	None	*d1, d2, e2, e3, e7, e8, e9, e12, s3, s4, s7, t2, t3*	None	*d1*	None	None
RO♀	*e2, e7, s3, ts*	None	None	None	None	*d1, e2, e7, e8, e9, e12, s3, s6, t2, t3*	*e2, e3, t3*	None	*e2*	*e11*
RO♂	*d1, d2, e2, e3, e8, e9, e10, e11, e12, s4, t2*	None	None	None	None	None	None	*s6*	None	None

*“↑” means upregulation, “↓” means downregulation, none means no genes changed after treatment in this tissue. GST expression was normalized to the transcription level geometric mean of β-actin and CYP4G55. The 2^–ΔΔCt^ and SE values were log_2_ transformed for plotting. Statistically significant differences in gene expression are shown in Supplementary Table S7.*

## Discussion

We successfully obtained sixteen cDNA gene sequences belonging to GSTs from the transcriptome of *D. armandi*. The gene sequences of the obtained GSTs were divided into four families (delta, epsilon, sigma, and theta) by phylogenetic analysis. This is consistent with the GST classification of other insects (Liu et al., 2017). Delta and epsilon are two different GST families in insects, and the GSTs that belong to these two families have the same metabolic function as pesticides (Ketterman et al., 2011). The GSTs in epsilon and delta are insect-specific which have large species-specific members in Coleoptera species like *Tribolium castaneum* (Shi et al., 2012). Further study also shows that delta and epsilon class GSTs are specifically involved in insecticide resistance in *Meligethes aeneus* to the most commonly used pyrethroids (Erban and Stara, 2014). Thus, the GST genes of these two families are also closely related to insect resistance to pesticides because the expression levels of the GSTs in the delta and epsilon families are upregulated in insecticide-resistant pests (Li et al., 2007). *D. armandi* has two delta-family GSTs and eight epsilon-family *DaGSTs*; these *DaGSTs* may also metabolize pesticides and other harmful compounds.

The resin present in conifers is the most important component of the chemical defense against the threat of foreign organisms. The resin secreted by a tree can inhibit beetle invasion, slow the invasion speed, and even repel the beetles with its detection (Erbilgin et al., 2003). Moreover, the monoterpene in the resin has toxic effects on the pest and causes debility or death through the respiratory system (Smith, 1965; López et al., 2011). The dispersal flight of the *Dendroctonus pseudotsugae* is oriented toward fresh wind-thrown and cut trees when these are present, while the beetle is attracted by oleoresin, particularly its α-pinene, camphene, and limonene fractions. The beetles are found to be repelled by *Pseudotsuga menziesii* resin and its fraction, including α-pinene and β-pinene, camphene, limonene, terpineol, and geraniol, when tested at close range in the laboratory (Rudinsky, 1966). And the toxicities of monoterpenes to bark beetles like *D. ponderosae* and *D. armandi* were performed with fumigation experiments (Reid and Purcell, 2011; Dai et al., 2015).

The experimental design and the theories behind the experiment were similar to those used in studies of detoxification enzymes (P450 enzymes, carboxylesterases, and GSTs) in *D. armandi* (Dai et al., 2014, 2015, 2016). The phloem of damaged conifers excretes volatile monoterpenoids, which can then enter the bodies of bark beetles through different routes. The chemical compounds enter the bark beetle body by penetrating the epidermis, accompanied by the invasion of its respiratory and digestive systems (Prates et al., 1998). Insect physiology conditions like life-stage and sex should be considered in studying GST enzymes activity under toxic stress (Wilczek et al., 2003). The strategy of this experiment is in consideration of not only how beetles intake terpenoids (feeding or stimulation), but also the different development stages of bark beetles. Therefore, the feeding experiments reflect GST gene function in the insect digestive and respiratory system, but the fumigation treatments only show GST gene function in the insect respiratory system. And the expression of GSTs in the different sexes of adult beetles was compared as a different behavior in host invasion.

The female adults had a higher expression of GSTs especially after feeding on host trees than males and other development stages under field conditions. And feeding with phloem of the host also induced more GSTs in females compared with males. These all indicated that GST genes were involved in the host selection of female adults and the multiple chemical compounds during host tree colonization (Zhang et al., 2010). The significantly increased transcript and protein accumulation of the *DpGSTs* gene with RNA-seq and proteomics after *D. ponderosae* fed on conifer phloem tissue (Robert et al., 2013; Pitt et al., 2014). Furthermore, when different developmental *D. armandi* were exposed to terpenoids, the expression levels of GSTs were more sensitive in adults than larvae and pupae, which was in accord with the higher transcription level in adults at field conditions. But different GST genes responded to different stimuli. Thus, beetles adapted to adverse environmental conditions such as multiple detoxification enzymes replying to multiple host chemical metabolites (Sheehan et al., 2001; López et al., 2013).

Previous studies on Chinese white pine beetle GSTs has shown the varying response to different stimuli (Dai et al., 2016). In the current study, the transcriptional level of *DaGSTs* in females had a stronger reaction than that in males after being treated with terpenoids, as female beetles are more susceptible to volatile substances in conifers (Zhang et al., 2010). Other studies have also shown that the transcriptional expression level of GSTs is overexpressed after exposure to monoterpenes, indicating that these substances can induce the metabolism of GSTs *in vivo*. In *Spodoptera eridania*, the significant upregulation of GSTs after feeding on monoterpenes (α-pinene, β-pinene, limonene, α-terpinene, and γ-terpinene) indicated that these substances induced the activity and expression level of GSTs (Brattsten et al., 1984; Gunderson et al., 1986).

Considering that different GST families have been reported to have different responses to monoterpenes in beetles, epsilon and sigma *DaGSTs* were differentially regulated in the different feeding and terpenoids treatment (Dai et al., 2016). This time, more differences were seen between sex in the *DaGST* transcript levels, and the regulation of some *DaGSTs* showed stimuli specifics with terpenoids treatment. GST gene transcription was more sensitive in females to monoterpenes than that in males, but 3-carene was a special stimulus to males which caused extensive overexpression of GSTs. (+)-3-carene lead to higher male beetle mortality in fumigant toxicity tests than other terpenoids (Dai et al., 2015), and also had a greater impact on the transcription of most esterase genes (Dai et al., 2019). The higher expression levels of GSTs in females had a relationship with the resistance to host chemicals which might be also involved in female oviposition.

As the expression profiles of GSTs in different tissues of Chinese white pine beetle shows some sex or tissue specifics. The overexpression of some GSTs in female antennae after being treated with monoterpenes indicates odor-degrading enzyme (ODE) functions (Dickens et al., 1992; Wojtasek and Leal, 1999; Maïbèche-Coisne et al., 2004). The results indicate that female insects first drill into conifers and their antennae need to metabolize various chemicals released by the host trees to avoid persistent excitation. Exotic pests can also use these chemicals to synthesize hormones or pheromones for population regulation (Byers, 1995). α-pinene has the highest proportion of volatile monoterpenes obtained from the resin of *P. armandi* (Huo and Chen, 2010). This is why many *DaGSTs* were overexpressed after exposure to α-pinene in the female gut, as the female gut participates in the detoxification process (Kasai et al., 2000; Tartar et al., 2009) and then transforms α-pinene to corresponding pheromones via intestinal bacteria present in some beetles (Kleinhentz et al., 1999; Seybold et al., 2000). The significant overexpression of many *DaGSTs* in male reproductive organs after being treated with α-pinene also demonstrates that the beetles convert tree monoterpenes into corresponding substances for population regulations (Zhao et al., 2017).

In conclusion, the *DaGST* genes belonging to different families participate in different metabolic processes in beetles and have different functional characteristics. It was confirmed that four families of GSTs in *D. armandi* were involved in the detoxification of terpenoids produced by host trees. The involvement in the metabolism of foreign substances was through different response patterns in both the feeding and stimulation treatments. The gene expression levels of GSTs were overexpressed in female beetles after being fed on host phloem which indicates that females have higher resistance to host chemical defenses because of their behaviors such as building galleries for oviposition. Moreover, in combination with the study of oxidative stress caused by conifer steroids, the current study aids in the understanding of the overcoming strategy of *D. armandi* for host chemical defense.

## Data Availability Statement

The datasets presented in this study can be found in online repositories. The names of the repository/repositories and accession number(s) can be found in the article/Supplementary Material.

## Author Contributions

HG, LD, and HC contributed to conceptualization. HG, DF, and YS performed the experiments and investigation. HG analyzed the data and wrote the manuscript. All authors have read and agreed to the published version of the manuscript.

## Conflict of Interest

The authors declare that the research was conducted in the absence of any commercial or financial relationships that could be construed as a potential conflict of interest.

## Footnotes

1 https://www.ncbi.nlm.nih.gov

2 https://www.ncbi.nlm.nih.gov/orffinder

3 https://www.rcsb.org

4 https://www.cbs.dtu.dk/services/TargetP
