# Supplementary material for: Isolation, Expression Profiling, and Regulation via Host Allelochemicals of 16 Glutathione S-Transferases in the Chinese White Pine Beetle, Dendroctonus armandi
Source: Front Physiol. 2020 Nov 12;11:546592. doi: 10.3389/fphys.2020.546592 (PMC7689161; doi:10.3389/fphys.2020.546592)
Supplement: Supplementary file 1 [file Table_1.DOCX]

Supplementary material

Table 1a. Primers used for amplification of the DaGSTs coding region

| **Gene** | **Sequence(5’ to 3’)** | **Primer purpose** |
| --- | --- | --- |
| *DaGSTd1* | F-GAGCAGCATTACTGACGG | cDNA cloning |
|  | R-TGCCACGATACTTGAGAC |  |
| *DaGSTd2* | F-TGGGAAAGTCGTGCTATT |  |
|  | R-ATCCATCTTCGTTTGCTT |  |
| *DaGSTe2* | F-ACACCGTACCCACCCTAG |  |
|  | R-TTGGCTCTTTCTGACCACTTTA |  |
| *DaGSTe3* | F-CACGCCATCAACTCCTAT |  |
|  | R-TACTTCGACACCCTTTGC |  |
| *DaGSTe7* | F-TTCTGGCAGACAGTCATG |  |
|  | R-TTCGCAGTTGTAAATCGT |  |
| *DaGSTe8* | F-ATGAGGGATTTATAGTATGG |  |
|  | R-AGATTTAAGCTGCGAGTA |  |
| *DaGSTe9* | F-AGAAGCAAGTGACTGTATG |  |
|  | R-AATTAAAGGCACTTCATG |  |
| *DaGSTe10* | F-AATTAAAGGCACTTCATG |  |
|  | R-ATATTTGTCTGGAGTGGG |  |
| *DaGSTe11* | F-ATAGTCACGCCATCAACG |  |
|  | R-TTAAGCCAGCAGCATTTA |  |
| *DaGSTe12* | F-TATGGCAAAGACGACAGT |  |
|  | R-TAATTCCACCAAAGCATC |  |
| *DaGSTs3* | F-GCAGAGCCAATAAGGTAT |  |
|  | R-AAACTAAATCAGCCCAAG |  |
| *DaGSTs4* | F-CTCCTCCCTACAAACTTACA |  |
|  | R-AACTTTCCTCCAACCAAG |  |
| *DaGSTs5* | F-ATGCCCTCCTCCACCTCCTA |  |
|  | R-GACCTTCTTGCCCACCAG |  |
| *DaGSTs6* | F-TATTTGCGTATGCTGGTC |  |
|  | R-TAAAGCGGTACTGCGATC |  |
| *DaGSTs7* | F-GATAACCGAGTTTCAAGGG |  |
|  | R-TTCGGAAGTGACAGGACA |  |
| *DaGSTt2* | F-GGCGAGTCCTCCGTCTTT |  |
|  | R-CTCCTGCTTGAACGTGGC |  |
| *DaGSTt3* | F-TCCTCCTTCGTTGGCAGTT |  |
|  | R-TGATGGCTTCCAGGCACA |  |

F= forward primer, R= reverse primer.

Supplementary material

Table 1b. Primers used for amplification of full-length DaGSTs

| **Gene** | **3′ RACE Sequence(5’ to 3’)** | **5′ RACE Sequence(5’ to 3’)** |
| --- | --- | --- |
| *DaGSTd1* | GSP-TTGCTGCCTCAGTCTCAAGTATCGT | GSP-GATCCTTCCCGTACACTGTAGCT |
|  | NGSP-CCTCAGTCTCAAGTATCGTGGCA | NGSP-GCCAATGGCTTTTGCCGTCAGTA |
| *DaGSTd2* | GSP-GTACCCGAAAGATCCCAAGAAGA | GSP-CAGCGACATAAGTTTGGCCTTCC |
|  | NGSP-TGAAGTTGTCGGCTATGATCTAG | NGSP-TTGGCGGCATCTTTAGAAGCACC |
| *DaGSTe2* | GSP-CTGCCGGAGACCGTCTTACAATA | GSP-CCGGCAGTCCACTCTGTTCTTTC |
|  | NGSP-GCCGGAGACCGTCTTACAATAGC | NGSP-TGCTGTGCCGCTGTCGAAATGAA |
| *DaGSTe3* | GSP-CAGATGAAAGCAGATGTGCGAGAA | GSP-TGGGCACATACACTTCAGTGGTAGA |
|  | NGSP-CAGTATTAACGCAAAGGGTGTCG | NGSP-CTCGCACATCTGCTTTCATCTGGT |
| *DaGSTe7* | GSP-AAAACGAAGAGCCTTGATTGACC | GSP-CCACGGCTCCTTCTTCATCCTGT |
|  | NGSP-TCAAAGCACATAGTCGGAAACCA | NGSP-GAAACTCAGTCTCCCAGTTGTT |
| *DaGSTe8* | GSP-CCGATATTCAGCCGAAAGACCCT | GSP-ATGCGTTCTGAGCTATCATTGTCG |
|  | NGSP-ATGAGCGACTGCCTCAGACCAAT | NGSP-GTCTGAGGCAGTCGCTCATCCTT |
| *DaGSTe9* | GSP-CAACGGTGGAAGAAGTCGTATTT | GSP-TCCTCGAAGTATGCCAACTCTTT |
|  | NGSP-TATCGGAATGGTTCAATAAAGCC | NGSP-AGTGAGAACATCTCCCGCTATCC |
| *DaGSTe10* | GSP-CCAAAGAAGTACAAGAGCGAGCAA | GSP-CGATATTTCCCGTTTGGTCTTGG |
|  | NGSP-GCCATGCCTATTCTTAGCACCAT | NGSP-CAGTCTTCCCGAACTTATCCACC |
| *DaGSTe11* | GSP-GCAATGAACTTCTTTGACGCTGGAT | GSP-GCTCGGAGTCAACAGTAGTGGGT |
|  | NGSP-ACCGACACCCACTACTGTTGACT | NGSP-CTATGTAGGCGTTGATGGCGTGA |
| *DaGSTe12* | GSP-AGTGCCATTACTGAGTCCTACAT | GSP-GCGTCCCAGCTTTAATTCCACCA |
|  | NGSP-TAGACTACTACGAGGAGACCAATG | NGSP-TCCAACACCATTGGTCTCCTCGT |
| *DaGSTs3* | GSP-CAGGGATCAGATGACTGGGAAAT | GSP-CCCAGTCATCTGATCCCTGTAGC |
|  | NGSP-GCGGTAACTTGGGCTGATTTAGT | NGSP-TTTAACTGAGGCCACTCTTCTCG |
| *DaGSTs4* | GSP-CCAGATATTTAGCCAAGCAAGTC | GSP-TACGCTTCCTTCGCAGCTTCATC |
|  | NGSP-TTATGGATTTCCGCAGCAAGGTG | NGSP-CGAGGTTTCCATAGCTGAGGAGC |
| *DaGSTs5* | GSP-AGTTTGAGGACAACCGCATTAACAGA | GSP-CGAGAATTGGCAGCTTTCCGTAC |
|  | NGSP-TCCCAGCTAAAGAGCAAGTCCAAGTAC | NGSP-GTGAGCTTGTAGGAGGTGGAGGAG |
| *DaGSTs6* | GSP-CAGGGAAAGGATGACTGGGAAAT | GSP-CTGAACCGACGAGAACTCCTGAA |
|  | NGSP-CTTCAGGAGTTCTCGTCGGTTCA | NGSP-GCGATAATCCGCCAGTGCTTCCA |
| *DaGSTs7* | GSP-GAGGCGAACCCTGAAACCAAACA | GSP-CTGCAAGTCCCACTAAACGTCCT |
|  | NGSP-ATTGATATTCGGGCAAATGGACT | NGSP-ACCCAATCGGCCCTTGAAACTCG |
| *DaGSTt2* | GSP-ATCCGAAGGATCAAACCCTCTAC | GSP-CCTGCTTGAACGTGGCGTACCAT |
|  | NGSP-CGATCTTTCCACCATGTGCTTAG | NGSP-CCATCGTCGTCGATTAGCGGAAT |
| *DaGSTt3* | GSP-CATCTGCGACTATGCGGTCAATC | GSP-GATGGCTTCCAGGCACATTGTCG |
|  | NGSP-AAAAGACCCAGATTGCGTTGAGC | NGSP-GGATTGACCGCATAGTCGCAGAT |

GSP= gene-specific primer (GSP) as forward primer, NGSP= nest gene-specific primer (NGSP) as reverse primer.

Supplementary material

Table 1c. Primers used for full-length validation of DaGSTs

| **Gene** | **Sequence(5’ to 3’)** | **Primer purpose** |
| --- | --- | --- |
| *DaGSTd1* | F-CTAACTTTACACCATTTCCCCCA | full-length validation |
|  | R-TTCGTTGGCTCCAGTCTGATTCG |  |
| *DaGSTd2* | F-TCCACAACACACGATTCCTACTA |  |
|  | R-ATCCATCTTCGTTTGCTTCCTTA |  |
| *DaGSTe2* | F-ATTTAGTCCTCCTTGTAGGGCTG |  |
|  | R-GTTTGCTCACTACTTTCCATTTA |  |
| *DaGSTe3* | F-ACGCAACACTCGGAAGTCCAGCA |  |
|  | R-GTTTCTCGCACATCTGCTTTCAT |  |
| *DaGSTe7* | F-CGGAAGTCCACCATTTGGGGCTG |  |
|  | R-CGCAGTTGTAAATCGTTGAAATG |  |
| *DaGSTe8* | F-GGGAAGCCCACCAGTAAATGCGG |  |
|  | R-TAAGTTTAGTTTTCAATCCATCT |  |
| *DaGSTe9* | F-TATTAGTGGACGCAGATGAAGAC |  |
|  | R-GTTGCCCCATTTTATCTAACCCC |  |
| *DaGSTe10* | F-AGTTATGATGGTGATGAAAGTGC |  |
|  | R-AGTTTTCACACCTCAGCCGATTC |  |
| *DaGSTe11* | F-ATTGAAGAGTGTAGAGTTTGTAG |  |
|  | R-AAGCCAGCAGCATTTACTTTCGC |  |
| *DaGSTe12* | F-GCGGGATGCGGCAATCGGTCTCG |  |
|  | R-ATTCACATAGTTCCTTTGTCCTG |  |
| *DaGSTs3* | F-TGACATCAGATACAAACCGAGCA |  |
|  | R-TATTTTAGCGTTTGTGCTGTGGA |  |
| *DaGSTs4* | F-ATGTCTCCTCCCTACAAACTTAC |  |
|  | R-GCAAATAAATCTTTCTTCGCCAT |  |
| *DaGSTs5* | F-GCCCTCCTCCACCTCCTACAAGC |  |
|  | R-ACCAAGACATTTATTTCGCTACA |  |
| *DaGSTs6* | F-TGCGTATGCTGGTCAAGACTATG |  |
|  | R-TTCCAAATCGTCTTGTCTTCCTG |  |
| *DaGSTs7* | F-CTCACATACTTTGACTTTACTGG |  |
|  | R-CGTTTTTCCACCCACGCCTTGAT |  |
| *DaGSTt2* | F-AAAGTTTTACTCAACATTGGCGA |  |
|  | R-GTTCCAAAAGTTTCCAAAGTTCC |  |
| *DaGSTt3* | F-CAGACAAATACCTGAAGGACCAA |  |
|  | R-ATTATTCATCAAATGTCCAAGTA |  |

F= forward primer, R= reverse primer.

**Supplementary material**

**Table 1d.** Primers used for RT-qPCR of *DaGST*s

| **Gene** | **Sequence(5’ to 3’)** | **Efficiency(%)** | **TM(°C)** |
| --- | --- | --- | --- |
| *DaGSTd1* | F-GATTGTGGAACATTATATCCCCGAA | 93.1 | 59 |
|  | R-AACACTGCCACGATACTTGAGACTG |  |  |
| *DaGSTd2* | F-GGTGCTTCTAAAGATGCCGCCAAAA | 104.3 | 62 |
|  | R-AAGGGGCTAGATCATAGCCGACAA |  |  |
| *DaGSTe2* | F-ACAGCCATGCGATACTACCATTCTT | 107.5 | 61 |
|  | R-TGTTTAGCAGTGCGTACACCTCCAA |  |  |
| *DaGSTe3* | F-GCCACGCCATCAACTCCTATT | 94.6 | 60 |
|  | R-TGTTTCTCGCACATCTGCTTTCATC |  |  |
| *DaGSTe7* | F-CATGGCATTCAACCAGTTCAGTCAA | 99.6 | 61 |
|  | R-ACGGCTCCTTCTTCATCCTGTCTAT |  |  |
| *DaGSTe8* | F-GAGCGACTGCCTCAGACCAAT | 96.5 | 61 |
|  | R-TTAAGCTGCGAGTACCACTTCCTTA |  |  |
| *DaGSTe9* | F-AATCTCTGGGCCTATTGGAAGTGTT | 94.5 | 61 |
|  | R-CCCATTTTATCTAACCCCTCCTTGT |  |  |
| *DaGSTe10* | F-ACGATGGGTTTATTCTGTGGG | 93.1 | 53 |
|  | R-CTCGCTCTTGTACTTCTTTGG |  |  |
| *DaGSTe11* | F-GCCTACATAGTGGGCAAATATGGAA | 97.6 | 61 |
|  | R-CAACAGTAGTGGGTGTCGGTGAAGT |  |  |
| *DaGSTe12* | F-TCACTGGAGATAGTTTGACGATAGC | 92.8 | 58 |
|  | R-CACCAAAGCATCCAAGTTGTTCA |  |  |
| *DaGSTs3* | F-TGGATCTGCGGTAACTTGGGCTGA | 95.3 | 64 |
|  | R-GATGGAGGAGGGTCGCTCTTAGGTC |  |  |
| *DaGSTs4* | F-GATGTTCTTATGGATTTCCGCAGCA | 90.5 | 60 |
|  | R-CCAACCAAGTAACCCCCATTTTCT |  |  |
| *DaGSTs5* | F-TTGAGGACAACCGCATTAACAGA | 90.7 | 59 |
|  | R-CGACCTTGACGAAGAGCCA |  |  |
| *DaGSTs6* | F-TTAGTGGAAGCACTGGCGGATTATC | 99.7 | 62 |
|  | R-TCTGAACCGACGAGAACTCCTGAAG |  |  |
| *DaGSTs7* | F-AGGCGAACCCTGAAACCAAAC | 97.5 | 60 |
|  | R-CTCCATTAAGGCGGCGAAGTA |  |  |
| *DaGSTt2* | F-TGGAGGATTTGGATTATAGCAGTGG | 107.3 | 59 |
|  | R-GCTCTTTCTTTAACATCTTTCGGGT |  |  |
| *DaGSTt3* | F-ACAAATACCCGAAGGACCAAACTCT | 99.1 | 61 |
|  | R-TTGACCGCATAGTCGCAGATGTGTT |  |  |

F= forward primer, R= reverse primer.

**Supplementary material**

**Table 2.** One-way analysis of variance (ANOVA) in the expression levels of 16 GSTs genes at different developmental stages and in different sexes of adult (emerged&feeding) *Dendroctonus armandi*

| **Gene** | **df** | **All Stages** | | **df** | **Emerged Adults** | | **df** | **Feeding Adults** | |
| --- | --- | --- | --- | --- | --- | --- | --- | --- | --- |
|  |  | **F** | **Sig.** |  | **F** | **Sig.** |  | **F** | **Sig.** |
| *DaGSTd1* | 6 | 12.292 | **<0.001** | 1 | 2.563 | 0.140 | 1 | 170.142 | **<0.001** |
| *DaGSTd2* | 6 | 9.592 | **<0.001** | 1 | 7.124 | **0.024** | 1 | 35.953 | **<0.001** |
| *DaGSTe2* | 6 | 14.623 | **<0.001** | 1 | 1.107 | 0.318 | 1 | 31.109 | **<0.001** |
| *DaGSTe3* | 6 | 27.489 | **<0.001** | 1 | 0.304 | 0.593 | 1 | 131.132 | **<0.001** |
| *DaGSTe7* | 6 | 28.133 | **<0.001** | 1 | 0.465 | 0.511 | 1 | 53.956 | **<0.001** |
| *DaGSTe8* | 6 | 14.922 | **<0.001** | 1 | 21.558 | **0.001** | 1 | 16.221 | **0.002** |
| *DaGSTe9* | 6 | 21.268 | **<0.001** | 1 | 3.615 | 0.086 | 1 | 84.355 | **<0.001** |
| *DaGSTe10* | 6 | 34.924 | **<0.001** | 1 | 35.094 | **<0.001** | 1 | 90.819 | **<0.001** |
| *DaGSTe11* | 6 | 13.372 | **<0.001** | 1 | 0.014 | 0.909 | 1 | 12.009 | **0.006** |
| *DaGSTe12* | 6 | 32.010 | **<0.001** | 1 | 0.446 | 0.519 | 1 | 172.621 | **<0.001** |
| *DaGSTs3* | 6 | 25.061 | **<0.001** | 1 | 5.249 | **0.045** | 1 | 36.621 | **<0.001** |
| *DaGSTs4* | 6 | 10.252 | **<0.001** | 1 | 1.768 | 0.213 | 1 | 64.069 | **<0.001** |
| *DaGSTs6* | 6 | 8.042 | **<0.001** | 1 | 0.715 | 0.418 | 1 | 11.250 | **0.007** |
| *DaGSTs7* | 6 | 10.798 | **<0.001** | 1 | <0.001 | 0.986 | 1 | 41.028 | **<0.001** |
| *DaGSTt2* | 6 | 14.469 | **<0.001** | 1 | 0.150 | 0.707 | 1 | 52.601 | **<0.001** |
| *DaGSTt3* | 6 | 20.489 | **<0.001** | 1 | 2.160 | 0.172 | 1 | 43.479 | **<0.001** |

Note: *P* values in bold indicate significant effects.

**Supplementary material**

**Table 3.** One-way analysis of variance (ANOVA) of 16 GSTs gene expression levels after phloem feeding (12 and 24 h) in adult *Dendroctonus armandi*

| **Gene** | **df** | **Female（**♀**）** | | **Male（**♂**）** | |
| --- | --- | --- | --- | --- | --- |
|  |  | **F** | **Sig.** | **F** | **Sig.** |
| *DaGSTd1* | 2 | 54.507 | **<0.001** | 0.391 | 0.693 |
| *DaGSTd2* | 2 | 5.334 | **0.047** | 0.087 | 0.917 |
| *DaGSTe2* | 2 | 129.365 | **<0.001** | 0.500 | 0.630 |
| *DaGSTe3* | 2 | 30.304 | **0.001** | 0.162 | 0.854 |
| *DaGSTe7* | 2 | 23.604 | **0.001** | 0.015 | 0.985 |
| *DaGSTe8* | 2 | 37.603 | **<0.001** | 0.116 | 0.892 |
| *DaGSTe9* | 2 | 92.957 | **<0.001** | 0.271 | 0.772 |
| *DaGSTe10* | 2 | 190.773 | **<0.001** | 0.583 | 0.587 |
| *DaGSTe11* | 2 | 33.359 | **0.001** | 0.187 | 0.834 |
| *DaGSTe12* | 2 | 118.708 | **<0.001** | 0.014 | 0.987 |
| *DaGSTs3* | 2 | 33.813 | **0.001** | 0.011 | 0.989 |
| *DaGSTs4* | 2 | 13.439 | **0.006** | 0.365 | 0.708 |
| *DaGSTs6* | 2 | 178.588 | **<0.001** | 0.303 | 0.749 |
| *DaGSTs7* | 2 | 44.483 | **<0.001** | 0.136 | 0.876 |
| *DaGSTt2* | 2 | 39.540 | **<0.001** | 4.942 | 0.054 |
| *DaGSTt3* | 2 | 43.868 | **<0.001** | 0.359 | 0.712 |

Note: *P* values in bold indicate significant effects.

**Supplementary material**

**Table 4.** One-way analysis of variance (ANOVA) of 16 GSTs gene expression levels after terpenoid treatment (1000 ppm for 8 and 24 h) in different stages of *Dendroctonus armandi*

| **Gene** | **df** | **(−)-*α*-Pinene** | | **(−)-*β*-Pinene** | | **(+)-3-carene** | | **(±)-Limonene** | | **Turpentine** | |
| --- | --- | --- | --- | --- | --- | --- | --- | --- | --- | --- | --- |
| **Larvae** |  | **F** | **Sig.** | **F** | **Sig.** | **F** | **Sig.** | **F** | **Sig.** | **F** | **Sig.** |
| *DaGSTd1* | 2 | 3.659 | 0.091 | 2.319 | 0.179 | 2.050 | 0.210 | 5.579 | **0.043** | 0.797 | 0.493 |
| *DaGSTd2* | 2 | 9.350 | **0.014** | 5.625 | **0.042** | 2.353 | 0.176 | 3.727 | 0.089 | 0.432 | 0.668 |
| *DaGSTe2* | 2 | 0.180 | 0.840 | 4.017 | 0.078 | 0.543 | 0.607 | 3.714 | 0.089 | 0.995 | 0.423 |
| *DaGSTe3* | 2 | 7.409 | **0.024** | 6.755 | **0.029** | 2.110 | 0.202 | 26.184 | **0.001** | 4.080 | 0.076 |
| *DaGSTe7* | 2 | 3.809 | 0.086 | 7.353 | **0.024** | 0.990 | 0.425 | 8.483 | **0.018** | 4.005 | 0.079 |
| *DaGSTe8* | 2 | 8.791 | **0.016** | 3.771 | 0.087 | 1.620 | 0.274 | 21.210 | **0.002** | 2.203 | 0.192 |
| *DaGSTe9* | 2 | 4.979 | 0.053 | 10.996 | **0.010** | 1.439 | 0.309 | 0.322 | 0.736 | 2.432 | 0.168 |
| *DaGSTe10* | 2 | 1.949 | 0.223 | 2.229 | 0.189 | 0.878 | 0.463 | 1.228 | 0.357 | 2.555 | 0.158 |
| *DaGSTe11* | 2 | 0.432 | 0.668 | 4.129 | 0.075 | 0.232 | 0.800 | 2.525 | 0.160 | 1.345 | 0.329 |
| *DaGSTe12* | 2 | 7.891 | **0.021** | 1.681 | 0.263 | 3.454 | 0.100 | 7.348 | **0.024** | 6.179 | **0.035** |
| *DaGSTs3* | 2 | 4.770 | 0.058 | 5.944 | **0.038** | 2.533 | 0.159 | 18.686 | **0.003** | 7.698 | **0.022** |
| *DaGSTs4* | 2 | 8.586 | **0.017** | 3.664 | 0.091 | 3.406 | 0.103 | 4.166 | 0.073 | 0.696 | 0.535 |
| *DaGSTs6* | 2 | 0.648 | 0.556 | 1.262 | 0.349 | 1.219 | 0.360 | 1.564 | 0.284 | 12.510 | **0.007** |
| *DaGSTs7* | 2 | 3.914 | 0.082 | 2.268 | 0.185 | 2.036 | 0.211 | 5.937 | **0.038** | 3.527 | 0.097 |
| *DaGSTt2* | 2 | 0.877 | 0.463 | 0.293 | 0.756 | 2.077 | 0.206 | 1.709 | 0.259 | 1.203 | 0.364 |
| *DaGSTt3* | 2 | 0.594 | 0.582 | 2.564 | 0.157 | 1.547 | 0.287 | 2.740 | 0.143 | 0.121 | 0.888 |

**Pupae**

| *DaGSTd1* | 2 | 0.635 | 0.562 | 0.146 | 0.867 | 0.117 | 0.892 | 0.124 | 0.886 | 0.315 | 0.741 |
| --- | --- | --- | --- | --- | --- | --- | --- | --- | --- | --- | --- |
| *DaGSTd2* | 2 | 0.590 | 0.584 | 1.651 | 0.268 | 1.036 | 0.411 | 1.219 | 0.360 | 0.142 | 0.870 |
| *DaGSTe2* | 2 | 1.134 | 0.382 | 3.953 | 0.080 | 0.930 | 0.445 | 0.138 | 0.874 | 0.103 | 0.904 |
| *DaGSTe3* | 2 | 0.503 | 0.628 | 1.519 | 0.293 | 1.238 | 0.355 | 0.557 | 0.600 | 0.145 | 0.868 |
| *DaGSTe7* | 2 | 2.641 | 0.150 | 2.477 | 0.164 | 3.612 | 0.093 | 5.560 | **0.043** | 1.504 | 0.295 |
| *DaGSTe8* | 2 | 1.273 | 0.346 | 2.874 | 0.133 | 1.043 | 0.409 | 0.183 | 0.837 | 0.162 | 0.854 |
| *DaGSTe9* | 2 | 3.468 | 0.100 | 3.580 | 0.095 | 1.160 | 0.375 | 1.126 | 0.384 | 0.067 | 0.936 |
| *DaGSTe10* | 2 | 2.115 | 0.202 | 0.820 | 0.484 | 2.041 | 0.211 | 0.640 | 0.560 | 0.429 | 0.670 |
| *DaGSTe11* | 2 | 2.034 | 0.212 | 1.544 | 0.288 | 2.583 | 0.155 | 4.065 | 0.077 | 1.639 | 0.270 |
| *DaGSTe12* | 2 | 1.773 | 0.248 | 3.132 | 0.117 | 5.523 | **0.044** | 12.482 | **0.007** | 3.082 | 0.120 |
| *DaGSTs3* | 2 | 4.399 | 0.067 | 2.501 | 0.162 | 10.347 | **0.011** | 4.185 | 0.073 | 4.380 | 0.067 |
| *DaGSTs4* | 2 | 0.745 | 0.514 | 0.666 | 0.548 | 0.817 | 0.486 | 0.860 | 0.469 | 0.255 | 0.783 |
| *DaGSTs6* | 2 | 1.143 | 0.380 | 1.184 | 0.369 | 0.498 | 0.631 | 0.537 | 0.610 | 0.807 | 0.489 |
| *DaGSTs7* | 2 | 3.814 | 0.085 | 2.852 | 0.135 | 5.075 | 0.051 | 0.415 | 0.678 | 1.001 | 0.422 |
| *DaGSTt2* | 2 | 7.370 | **0.024** | 9.267 | **0.015** | 10.265 | **0.012** | 7.340 | **0.024** | 2.957 | 0.128 |
| *DaGSTt3* | 2 | 0.057 | 0.945 | 0.081 | 0.923 | 0.275 | 0.768 | 0.157 | 0.858 | 0.345 | 0.722 |

**Teneral adults**

| *DaGSTd1* | 2 | 19.603 | **0.002** | 37.983 | **<0.001** | 43.821 | **<0.001** | 47.817 | **<0.001** | 0.303 | 0.749 |
| --- | --- | --- | --- | --- | --- | --- | --- | --- | --- | --- | --- |
| *DaGSTd2* | 2 | 6.820 | **0.029** | 34.024 | **0.001** | 12.938 | **0.007** | 41.052 | **<0.001** | 2.552 | 0.158 |
| *DaGSTe2* | 2 | 0.779 | 0.500 | 1.421 | 0.312 | 8.733 | **0.017** | 15.634 | **0.004** | 1.139 | 0.381 |
| *DaGSTe3* | 2 | 12.696 | **0.007** | 18.743 | **0.003** | 43.893 | **<0.001** | 96.470 | **<0.001** | 4.949 | 0.054 |
| *DaGSTe7* | 2 | 20.768 | **0.002** | 51.703 | **<0.001** | 36.392 | **<0.001** | 69.618 | **<0.001** | 3.708 | 0.089 |
| *DaGSTe8* | 2 | 8.438 | **0.018** | 2.666 | 0.148 | 3.924 | 0.081 | 6.740 | **0.029** | 4.003 | 0.079 |
| *DaGSTe9* | 2 | 5.214 | **0.049** | 8.527 | **0.018** | 4.211 | 0.072 | 6.385 | **0.033** | 4.484 | 0.064 |
| *DaGSTe10* | 2 | 25.303 | **0.001** | 13.659 | **0.006** | 12.216 | **0.008** | 13.512 | **0.006** | 4.711 | 0.059 |
| *DaGSTe11* | 2 | 4.762 | 0.058 | 9.819 | **0.013** | 11.235 | **0.009** | 39.737 | **<0.001** | 0.810 | 0.488 |
| *DaGSTe12* | 2 | 6.781 | **0.029** | 10.529 | **0.011** | 12.969 | **0.007** | 13.174 | **0.006** | 3.628 | 0.093 |
| *DaGSTs3* | 2 | 27.228 | **0.001** | 67.763 | **<0.001** | 91.164 | **<0.001** | 28.394 | **0.001** | 4.117 | 0.075 |
| *DaGSTs4* | 2 | 20.333 | **0.002** | 41.304 | **<0.001** | 27.648 | **0.001** | 19.539 | **0.002** | 4.537 | 0.063 |
| *DaGSTs6* | 2 | 4.704 | 0.059 | 4.738 | 0.058 | 1.720 | 0.257 | 7.656 | **0.022** | 3.090 | 0.120 |
| *DaGSTs7* | 2 | 32.191 | **0.001** | 81.855 | **<0.001** | 56.285 | **<0.001** | 1.005 | 0.420 | 6.543 | **0.031** |
| *DaGSTt2* | 2 | 16.825 | **0.003** | 6.619 | **0.030** | 1.666 | 0.266 | 19.281 | **0.002** | 4.880 | 0.055 |
| *DaGSTt3* | 2 | 11.879 | **0.008** | 51.566 | **<0.001** | 2.876 | 0.133 | 31.982 | **0.001** | 11.729 | **0.008** |

**Emerged adult females (**♀**)**

| *DaGSTd1* | 2 | 92.861 | **<0.001** | 22.647 | **0.002** | 2.915 | 0.130 | 7.187 | **0.026** | 10.341 | **0.011** |
| --- | --- | --- | --- | --- | --- | --- | --- | --- | --- | --- | --- |
| *DaGSTd2* | 2 | 4.737 | 0.058 | 10.741 | **0.010** | 3.156 | 0.116 | 9.399 | **0.014** | 12.033 | **0.008** |
| *DaGSTe2* | 2 | 1.695 | 0.261 | 7.741 | **0.022** | 0.289 | 0.759 | 27.847 | **0.001** | 29.975 | **0.001** |
| *DaGSTe3* | 2 | 8.839 | **0.016** | 13.614 | **0.006** | 0.330 | 0.731 | 5.373 | **0.046** | 15.726 | **0.004** |
| *DaGSTe7* | 2 | 44.018 | **<0.001** | 10.362 | **0.011** | 0.061 | 0.941 | 10.271 | **0.012** | 6.747 | **0.029** |
| *DaGSTe8* | 2 | 41.688 | **<0.001** | 79.112 | **<0.001** | 1.826 | 0.240 | 32.412 | **0.001** | 36.386 | **<0.001** |
| *DaGSTe9* | 2 | 73.847 | **<0.001** | 49.361 | **<0.001** | 8.294 | **0.019** | 7.149 | **0.026** | 10.018 | **0.012** |
| *DaGSTe10* | 2 | 1.643 | 0.270 | 9.025 | **0.016** | 1.168 | 0.373 | 1.852 | 0.236 | 6.825 | **0.028** |
| *DaGSTe11* | 2 | 1.309 | 0.337 | 0.040 | 0.961 | 1.481 | 0.300 | 6.198 | **0.035** | 6.136 | **0.035** |
| *DaGSTe12* | 2 | 64.182 | **<0.001** | 19.129 | **0.002** | 3.666 | 0.091 | 18.452 | **0.003** | 2.848 | 0.135 |
| *DaGSTs3* | 2 | 4.206 | 0.072 | 3.224 | 0.112 | 0.633 | 0.563 | 9.342 | **0.014** | 15.826 | **0.004** |
| *DaGSTs4* | 2 | 31.159 | **0.001** | 12.231 | **0.008** | 2.327 | 0.179 | 13.110 | **0.006** | 8.856 | **0.016** |
| *DaGSTs6* | 2 | 9.731 | **0.013** | 6.188 | **0.035** | 4.056 | 0.077 | 6.357 | **0.033** | 12.000 | **0.008** |
| *DaGSTs7* | 2 | 0.160 | 0.856 | 3.237 | 0.111 | 2.032 | 0.212 | 22.807 | **0.002** | 27.051 | **0.001** |
| *DaGSTt2* | 2 | 116.449 | **<0.001** | 33.632 | **0.001** | 9.821 | **0.013** | 18.778 | **0.003** | 48.709 | **<0.001** |
| *DaGSTt3* | 2 | 18.542 | **0.003** | 21.046 | **0.002** | 6.027 | **0.037** | 9.544 | **0.014** | 20.424 | **0.002** |

**Emerged adult males (**♂**)**

| *DaGSTd1* | 2 | 1.225 | 0.358 | 2.698 | 0.146 | 5.292 | **0.047** | 0.815 | 0.486 | 0.991 | 0.425 |
| --- | --- | --- | --- | --- | --- | --- | --- | --- | --- | --- | --- |
| *DaGSTd2* | 2 | 2.098 | 0.204 | 2.156 | 0.197 | 15.524 | **0.004** | 1.103 | 0.391 | 0.052 | 0.949 |
| *DaGSTe2* | 2 | 5.585 | **0.043** | 0.912 | 0.451 | 77.190 | **<0.001** | 17.585 | **0.003** | 9.364 | **0.014** |
| *DaGSTe3* | 2 | 0.748 | 0.513 | 0.717 | 0.526 | 5.121 | 0.050 | 0.113 | 0.895 | 0.035 | 0.966 |
| *DaGSTe7* | 2 | 0.093 | 0.913 | 0.025 | 0.976 | 37.939 | **<0.001** | 2.436 | 0.168 | 0.309 | 0.745 |
| *DaGSTe8* | 2 | 20.489 | **0.002** | 14.312 | **0.005** | 10.673 | **0.011** | 4.423 | 0.066 | 8.658 | **0.017** |
| *DaGSTe9* | 2 | 13.439 | **0.006** | 24.417 | **0.001** | 22.777 | **0.002** | 3.537 | 0.097 | 1.119 | 0.386 |
| *DaGSTe10* | 2 | 8.845 | **0.016** | 14.612 | **0.005** | 10.726 | **0.010** | 112.578 | **<0.001** | 5.112 | 0.051 |
| *DaGSTe11* | 2 | 24.576 | **0.001** | 23.246 | **0.001** | 132.524 | **<0.001** | 6.367 | **0.033** | 17.919 | **0.003** |
| *DaGSTe12* | 2 | 7.855 | **0.021** | 4.267 | 0.070 | 32.119 | **0.001** | 1.698 | 0.260 | 2.128 | 0.200 |
| *DaGSTs3* | 2 | 1.413 | 0.314 | 1.171 | 0.372 | 58.293 | **<0.001** | 1.631 | 0.272 | 0.109 | 0.899 |
| *DaGSTs4* | 2 | 1.701 | 0.260 | 0.841 | 0.476 | 48.337 | **<0.001** | 1.238 | 0.355 | 0.041 | 0.960 |
| *DaGSTs6* | 2 | 19.655 | **0.002** | 0.720 | 0.524 | 155.287 | **<0.001** | 9.113 | **0.015** | 6.979 | **0.027** |
| *DaGSTs7* | 2 | 0.779 | 0.500 | 0.007 | 0.993 | 11.041 | **0.010** | 1.957 | 0.222 | 1.434 | 0.310 |
| *DaGSTt2* | 2 | 14.645 | **0.005** | 6.174 | **0.035** | 5.004 | 0.053 | 5.021 | 0.052 | 16.473 | **0.004** |
| *DaGSTt3* | 2 | 2.971 | 0.127 | 1.654 | 0.268 | 2.415 | 0.170 | 1.693 | 0.261 | 0.095 | 0.911 |

Note: *P* values in bold indicate significant effects.

**Supplementary material**

Table 5 Tukey test results among stimuli and exposure time of GSTs genes in different developmental stages expression from *Dendroctonus armandi*

**A Larvae**

| Gene | (−)-α-pinene | | | (−)-β-pinene | | | (+)-3-carene | | | (±)-Limonene | | | Turpentine | | |
| --- | --- | --- | --- | --- | --- | --- | --- | --- | --- | --- | --- | --- | --- | --- | --- |
|  | 0 h | 8 h | 24 h | 0 h | 8 h | 24 h | 0 h | 8 h | 24 h | 0 h | 8 h | 24 h | 0 h | 8 h | 24 h |
| *DaGSTd1* | a | a | a | a | a | a | a | a | a | ab | a | b | a | a | a |
| *DaGSTd2* | b | a | b | a | a | b | a | a | a | a | a | a | a | a | a |
| *DaGSTe2* | a | a | a | a | a | a | a | a | a | a | a | a | a | a | a |
| *DaGSTe3* | ab | a | b | ab | a | b | a | a | a | a | a | b | a | a | a |
| *DaGSTe7* | a | a | a | b | a | b | a | a | a | ab | a | b | a | a | a |
| *DaGSTe8* | ab | a | b | a | a | a | a | a | a | b | a | b | a | a | a |
| *DaGSTe9* | a | a | a | b | a | b | a | a | a | a | a | a | a | a | a |
| *DaGSTe10* | a | a | a | a | a | a | a | a | a | a | a | a | a | a | a |
| *DaGSTe11* | a | a | a | a | a | a | a | a | a | a | a | a | a | a | a |
| *DaGSTe12* | ab | a | b | a | a | a | a | a | a | ab | a | b | a | b | ab |
| *DaGSTs3* | a | a | a | ab | a | b | a | a | a | a | a | b | a | b | ab |
| *DaGSTs4* | ab | a | b | a | a | a | a | a | a | a | a | a | a | a | a |
| *DaGSTs6* | a | a | a | a | a | a | a | a | a | a | a | a | a | b | ab |
| *DaGSTs7* | a | a | a | a | a | a | a | a | a | ab | a | b | a | a | a |
| *DaGSTt2* | a | a | a | a | a | a | a | a | a | a | a | a | a | a | a |
| *DaGSTt3* | a | a | a | a | a | a | a | a | a | a | a | a | a | a | a |

**B Pupae**

| Gene | (−)-α-pinene | | | (−)-β-pinene | | | (+)-3-carene | | | (±)-Limonene | | | Turpentine | | |
| --- | --- | --- | --- | --- | --- | --- | --- | --- | --- | --- | --- | --- | --- | --- | --- |
|  | 0 h | 8 h | 24 h | 0 h | 8 h | 24 h | 0 h | 8 h | 24 h | 0 h | 8 h | 24 h | 0 h | 8 h | 24 h |
| *DaGSTd1* | a | a | a | a | a | a | a | a | a | a | a | a | a | a | a |
| *DaGSTd2* | a | a | a | a | a | a | a | a | a | a | a | a | a | a | a |
| *DaGSTe2* | a | a | a | a | a | a | a | a | a | a | a | a | a | a | a |
| *DaGSTe3* | a | a | a | a | a | a | a | a | a | a | a | a | a | a | a |
| *DaGSTe7* | a | a | a | a | a | a | a | a | a | b | a | ab | a | a | a |
| *DaGSTe8* | a | a | a | a | a | a | a | a | a | a | a | a | a | a | a |
| *DaGSTe9* | a | a | a | a | a | a | a | a | a | a | a | a | a | a | a |
| *DaGSTe10* | a | a | a | a | a | a | a | a | a | a | a | a | a | a | a |
| *DaGSTe11* | a | a | a | a | a | a | a | a | a | a | a | a | a | a | a |
| *DaGSTe12* | a | a | a | a | a | a | b | a | ab | b | a | b | a | a | a |
| *DaGSTs3* | a | a | a | a | a | a | b | a | a | a | a | a | a | a | a |
| *DaGSTs4* | a | a | a | a | a | a | a | a | a | a | a | a | a | a | a |
| *DaGSTs6* | a | a | a | a | a | a | a | a | a | a | a | a | a | a | a |
| *DaGSTs7* | a | a | a | a | a | a | a | a | a | a | a | a | a | a | a |
| *DaGSTt2* | ab | a | b | ab | a | b | ab | a | b | b | a | b | a | a | a |
| *DaGSTt3* | a | a | a | a | a | a | a | a | a | a | a | a | a | a | a |

**C Teneral adults**

| Gene | (−)-α-pinene | | | (−)-β-pinene | | | (+)-3-carene | | | (±)-Limonene | | | Turpentine | | |
| --- | --- | --- | --- | --- | --- | --- | --- | --- | --- | --- | --- | --- | --- | --- | --- |
|  | 0 h | 8 h | 24 h | 0 h | 8 h | 24 h | 0 h | 8 h | 24 h | 0 h | 8 h | 24 h | 0 h | 8 h | 24 h |
| *DaGSTd1* | a | b | b | a | c | b | a | c | b | a | c | b | a | a | a |
| *DaGSTd2* | a | ab | b | a | b | b | a | b | b | a | c | b | a | a | a |
| *DaGSTe2* | a | a | a | a | a | a | a | b | ab | a | b | b | a | a | a |
| *DaGSTe3* | a | b | b | a | b | b | a | c | b | a | c | b | a | a | a |
| *DaGSTe7* | a | b | b | a | b | b | a | b | b | a | c | b | a | a | a |
| *DaGSTe8* | ab | a | b | a | a | a | a | a | a | a | ab | b | a | a | a |
| *DaGSTe9* | ab | a | b | ab | a | b | a | a | a | a | ab | b | a | a | a |
| *DaGSTe10* | a | a | b | a | a | b | a | a | b | a | b | a | a | a | a |
| *DaGSTe11* | a | a | a | a | b | ab | a | b | b | a | c | b | a | a | a |
| *DaGSTe12* | a | ab | b | a | b | b | a | b | b | a | b | b | a | a | a |
| *DaGSTs3* | a | b | b | a | c | b | a | c | b | a | b | b | a | a | a |
| *DaGSTs4* | a | b | c | a | b | b | a | b | b | a | b | b | a | a | a |
| *DaGSTs6* | a | a | a | a | a | a | a | a | a | a | a | b | a | a | a |
| *DaGSTs7* | a | c | b | a | c | b | a | c | b | a | a | a | a | b | ab |
| *DaGSTt2* | b | a | b | ab | a | b | a | a | a | a | b | b | ab | a | b |
| *DaGSTt3* | b | ab | a | c | b | a | a | a | a | a | b | a | b | a | ab |

**D Emerged adults**

| Gene | Sexes | (−)-α-pinene | | | (−)-β-pinene | | | (+)-3-carene | | | (±)-Limonene | | | Turpentine | | |
| --- | --- | --- | --- | --- | --- | --- | --- | --- | --- | --- | --- | --- | --- | --- | --- | --- |
|  |  | 0 h | 8 h | 24 h | 0 h | 8 h | 24 h | 0 h | 8 h | 24 h | 0 h | 8 h | 24 h | 0 h | 8 h | 24 h |
| *DaGSTd1* | ♀ | a | a | b | a | a | b | a | a | a | ab | b | a | b | a | a |
|  | ♂ | a | a | a | a | a | a | b | b | a | a | a | a | a | a | a |
| *DaGSTd2* | ♀ | a | a | a | a | a | b | a | a | a | ab | b | a | b | ab | a |
|  | ♂ | a | a | a | a | a | a | b | b | a | a | a | a | a | a | a |
| *DaGSTe2* | ♀ | a | a | a | ab | a | b | a | a | a | b | b | a | b | b | a |
|  | ♂ | ab | b | a | a | a | a | b | b | a | b | b | a | ab | b | a |
| *DaGSTe3* | ♀ | a | a | b | ab | a | b | a | a | a | a | a | a | b | a | a |
|  | ♂ | a | a | a | a | a | a | a | a | a | a | a | a | a | a | a |
| *DaGSTe7* | ♀ | b | a | c | ab | a | b | a | a | a | b | b | a | b | ab | a |
|  | ♂ | a | a | a | a | a | a | b | b | a | a | a | a | a | a | a |
| *DaGSTe8* | ♀ | a | b | c | a | a | b | a | a | a | a | b | a | c | b | a |
|  | ♂ | a | c | b | a | b | a | b | b | a | a | a | a | a | b | b |
| *DaGSTe9* | ♀ | a | b | c | a | a | b | a | ab | b | a | b | b | a | ab | b |
|  | ♂ | a | b | b | a | a | b | b | b | a | a | a | a | a | a | a |
| *DaGSTe10* | ♀ | a | a | a | a | b | b | a | a | a | a | a | a | b | b | a |
|  | ♂ | b | a | a | b | a | a | b | b | a | b | b | a | a | a | a |
| *DaGSTe11* | ♀ | a | a | a | a | a | a | a | a | a | ab | b | a | ab | b | a |
|  | ♂ | b | b | a | a | b | a | b | b | a | ab | b | a | b | b | a |
| *DaGSTe12* | ♀ | a | b | c | a | a | b | a | a | a | a | b | a | a | a | a |
|  | ♂ | a | b | ab | a | a | a | b | b | a | a | a | a | a | a | a |
| *DaGSTs3* | ♀ | a | a | a | a | a | a | a | a | a | b | b | a | b | a | a |
|  | ♂ | a | a | a | a | a | a | b | b | a | a | a | a | a | a | a |
| *DaGSTs4* | ♀ | a | b | b | a | ab | b | a | a | a | a | b | a | b | ab | a |
|  | ♂ | a | a | a | a | a | a | b | b | a | a | a | a | a | a | a |
| *DaGSTs6* | ♀ | ab | b | a | ab | a | b | a | a | a | ab | b | a | b | ab | a |
|  | ♂ | b | b | a | a | a | a | b | c | a | b | b | a | b | ab | a |
| *DaGSTs7* | ♀ | a | a | a | a | a | a | a | a | a | b | b | a | b | b | a |
|  | ♂ | a | a | a | a | a | a | b | b | a | a | a | a | a | a | a |
| *DaGSTt2* | ♀ | a | b | c | a | a | b | a | ab | b | a | a | b | a | a | b |
|  | ♂ | a | b | b | a | ab | b | a | a | a | a | a | a | a | a | b |
| *DaGSTt3* | ♀ | b | a | b | b | a | b | b | a | ab | b | a | a | b | a | a |
|  | ♂ | a | a | a | a | a | a | a | a | a | a | a | a | a | a | a |

Note: Different letters indicate significant differences at *P <*0.05 (Tukey test) of each stimulus at exposure times of 0, 8 and 24 h. Cells of different colors indicate the different trends in GSTs with each stimulus at exposure times of 0, 8 and 24 h: blue = downregulation, red = upregulation, and green= opposite trends at 8 and 24 h.

| **Supplementary material**  **Table 6.** Two-way analysis of variance (ANOVA) of 16 GSTs gene expression levels in different sexes and tissues in emerged adult of *Dendroctonus armandi* | | | | | | | | | |
| --- | --- | --- | --- | --- | --- | --- | --- | --- | --- |
| Gene | Sex | | | Tissue | | | Sex*Tissue | | |
|  | df | F | P | df | F | P | df | F | P |
| DaGSTd1 | 1 | 1.842 | 0.200 | 2 | 6.737 | **0.011** | 2 | 2.212 | 0.152 |
| DaGSTd2 | 1 | 3.880 | 0.072 | 2 | 0.462 | 0.641 | 2 | 1.398 | 0.285 |
| DaGSTe2 | 1 | 8.858 | **0.012** | 2 | 8.047 | **0.006** | 2 | 4.356 | **0.038** |
| DaGSTe3 | 1 | 0.591 | 0.457 | 2 | 11.924 | **0.001** | 2 | 1.011 | 0.393 |
| DaGSTe7 | 1 | 2.942 | 0.112 | 2 | 9.727 | **0.003** | 2 | 2.296 | 0.143 |
| DaGSTe8 | 1 | 13.174 | **0.003** | 2 | 0.706 | 0.513 | 2 | 14.431 | **0.001** |
| DaGSTe9 | 1 | 3.800 | 0.075 | 2 | 8.983 | **0.004** | 2 | 0.947 | 0.415 |
| DaGSTe10 | 1 | 27.572 | **<0.001** | 2 | 5.466 | **0.021** | 2 | 4.835 | **0.029** |
| DaGSTe11 | 1 | 1.908 | 0.192 | 2 | 32.483 | **<0.001** | 2 | 0.290 | 0.754 |
| DaGSTe12 | 1 | 1.542 | 0.238 | 2 | 10.844 | **0.002** | 2 | 4.138 | **0.043** |
| DaGSTs3 | 1 | 3.694 | 0.079 | 2 | 8.566 | **0.005** | 2 | 0.439 | 0.654 |
| DaGSTs4 | 1 | 6.897 | **0.022** | 2 | 6.436 | **0.013** | 2 | 1.042 | 0.383 |
| DaGSTs6 | 1 | 6.115 | **0.029** | 2 | 7.624 | **0.007** | 2 | 1.794 | 0.208 |
| DaGSTs7 | 1 | 3.082 | 0.105 | 2 | 12.550 | **0.001** | 2 | 0.852 | 0.451 |
| DaGSTt2 | 1 | 16.873 | **0.001** | 2 | 19.453 | **<0.001** | 2 | 3.008 | 0.087 |
| DaGSTt3 | 1 | 2.398 | 0.147 | 2 | 11.601 | **0.002** | 2 | 0.187 | 0.831 |

Note: *P* values in bold indicate significant difference between sexes, tissues and S*T interaction with Two-way ANOVA.

**Supplementary material**

**Table 7.** One-way analysis of variance (ANOVA) of 16 GSTs gene expression levels after terpenoid treatment (1000 ppm for 8 h) in different tissues of *Dendroctonusarmandi*

| **Gene** | **df** | **Tissue** | **(–)-*α*-Pinene** | | **(–)-*β*-Pinene** | | **(+)-3-carene** | | **(±)-Limonene** | | **Turpentine** | |
| --- | --- | --- | --- | --- | --- | --- | --- | --- | --- | --- | --- | --- |
|  |  |  | **F** | **Sig.** | **F** | **Sig.** | **F** | **Sig.** | **F** | **Sig.** | **F** | **Sig.** |
| *DaGSTd1* | 1 | A♀ | 3.390 | 0.139 | 0.636 | 0.470 | 1.913 | 0.239 | 0.139 | 0.728 | 1.315 | 0.315 |
|  | 1 | A♂ | 0.609 | 0.479 | 1.547 | 0.282 | 2.870 | 0.166 | 1.648 | 0.269 | 1.521 | 0.285 |
|  | 1 | G♀ | 10.100 | **0.034** | 3.414 | 0.138 | 1.137 | 0.346 | 0.473 | 0.529 | 0.035 | 0.860 |
|  | 1 | G♂ | 0.080 | 0.791 | 4.151 | 0.111 | 46.829 | **0.002** | 33.784 | **0.004** | 5.232 | 0.084 |
|  | 1 | RO♀ | 2.204 | 0.212 | 0.554 | 0.498 | 34.960 | **0.004** | 0.058 | 0.821 | 1.636 | 0.270 |
|  | 1 | RO♂ | 17.398 | **0.014** | 0.743 | 0.437 | 4.050 | 0.114 | 0.561 | 0.496 | 1.690 | 0.263 |
| *DaGSTd2* | 1 | A♀ | 2.618 | 0.181 | 0.703 | 0.449 | 1.765 | 0.255 | 0.032 | 0.868 | 6.780 | 0.060 |
|  | 1 | A♂ | 0.282 | 0.623 | 0.699 | 0.450 | 1.255 | 0.325 | 0.897 | 0.397 | 0.692 | 0.452 |
|  | 1 | G♀ | 0.831 | 0.414 | 0.795 | 0.423 | 0.066 | 0.809 | 0.048 | 0.838 | 2.039 | 0.227 |
|  | 1 | G♂ | <0.001 | 0.999 | 7.982 | **0.048** | 31.447 | **0.005** | 0.144 | 0.723 | 4.590 | 0.099 |
|  | 1 | RO♀ | 5.822 | 0.073 | 0.004 | 0.954 | 5.842 | 0.073 | 2.377 | 0.198 | 3.105 | 0.153 |
|  | 1 | RO♂ | 33.083 | **0.005** | 0.588 | 0.486 | 0.721 | 0.444 | 1.601 | 0.274 | 0.153 | 0.716 |
| *DaGSTe2* | 1 | A♀ | 0.775 | 0.429 | <0.001 | 0.987 | 3.010 | 0.158 | 0.047 | 0.839 | 0.614 | 0.477 |
|  | 1 | A♂ | 0.412 | 0.556 | 0.461 | 0.534 | 0.760 | 0.433 | 1.141 | 0.346 | 0.440 | 0.543 |
|  | 1 | G♀ | 2.615 | 0.181 | 1.611 | 0.273 | 1.389 | 0.304 | 1.660 | 0.267 | 0.095 | 0.773 |
|  | 1 | G♂ | 0.016 | 0.904 | 3.496 | 0.135 | 45.390 | **0.003** | 0.240 | 0.650 | 0.758 | 0.433 |
|  | 1 | RO♀ | 19.256 | **0.012** | 1.312 | 0.316 | 39.848 | **0.003** | 10.455 | **0.032** | 8.360 | **0.045** |
|  | 1 | RO♂ | 14.957 | **0.018** | 0.739 | 0.438 | 3.609 | 0.130 | 0.034 | 0.862 | 1.787 | 0.252 |
| *DaGSTe3* | 1 | A♀ | 2.716 | 0.175 | 0.060 | 0.819 | 1.819 | 0.249 | 0.368 | 0.577 | 0.863 | 0.406 |
|  | 1 | A♂ | 0.177 | 0.696 | 0.482 | 0.526 | 1.101 | 0.353 | 0.902 | 0.396 | 0.703 | 0.449 |
|  | 1 | G♀ | 33.169 | **0.005** | 0.147 | 0.721 | 0.798 | 0.422 | 0.347 | 0.588 | 0.809 | 0.419 |
|  | 1 | G♂ | 3.816 | 0.122 | 1.686 | 0.264 | 38.428 | **0.003** | 0.317 | 0.603 | 1.411 | 0.301 |
|  | 1 | RO♀ | 6.047 | 0.070 | 0.034 | 0.863 | 2.859 | 0.166 | 12.360 | **0.025** | 0.633 | 0.471 |
|  | 1 | RO♂ | 17.498 | **0.014** | 0.325 | 0.599 | 0.525 | 0.509 | 1.620 | 0.272 | 0.004 | 0.955 |
| *DaGSTe7* | 1 | A♀ | 2.245 | 0.208 | 0.227 | 0.658 | 2.730 | 0.174 | 5.674 | 0.076 | 3.382 | 0.140 |
|  | 1 | A♂ | 0.547 | 0.501 | 1.246 | 0.327 | 2.507 | 0.189 | 1.609 | 0.273 | 0.976 | 0.379 |
|  | 1 | G♀ | 5.762 | 0.074 | 1.085 | 0.356 | 2.011 | 0.229 | 0.184 | 0.690 | 0.584 | 0.487 |
|  | 1 | G♂ | 3.265 | 0.145 | 2.656 | 0.178 | 145.083 | **<0.001** | 0.079 | 0.793 | 2.071 | 0.224 |
|  | 1 | RO♀ | 10.049 | **0.034** | 2.445 | 0.193 | 136.079 | **<0.001** | 3.232 | 0.147 | 0.021 | 0.891 |
|  | 1 | RO♂ | 5.200 | 0.085 | 0.696 | 0.451 | 4.821 | 0.093 | 0.586 | 0.487 | <0.001 | 0.984 |
| *DaGSTe8* | 1 | A♀ | 11.547 | **0.027** | 17.272 | **0.014** | 1.607 | 0.274 | 2.591 | 0.183 | 13.234 | **0.022** |
|  | 1 | A♂ | 0.184 | 0.690 | 0.646 | 0.467 | 1.238 | 0.328 | 0.789 | 0.425 | 0.735 | 0.440 |
|  | 1 | G♀ | 23.505 | **0.008** | 0.245 | 0.647 | 0.307 | 0.609 | 18.507 | **0.013** | 4.070 | 0.114 |
|  | 1 | G♂ | 3.708 | 0.126 | 7.289 | 0.054 | 34.166 | **0.004** | 0.114 | 0.753 | 0.033 | 0.864 |
|  | 1 | RO♀ | 2.779 | 0.171 | 0.940 | 0.387 | 107.896 | **<0.001** | 0.452 | 0.538 | 0.679 | 0.456 |
|  | 1 | RO♂ | 14.668 | **0.019** | 1.316 | 0.315 | 1.482 | 0.290 | 0.416 | 0.554 | 0.481 | 0.526 |
| *DaGSTe9* | 1 | A♀ | 5.738 | 0.075 | 5.351 | 0.082 | 2.883 | 0.165 | 0.549 | 0.500 | 15.323 | **0.017** |
|  | 1 | A♂ | 0.072 | 0.801 | 0.372 | 0.575 | 1.174 | 0.340 | 1.455 | 0.294 | 0.258 | 0.638 |
|  | 1 | G♀ | 76.865 | **0.001** | 1.239 | 0.328 | 0.901 | 0.396 | 3.648 | 0.129 | 3.808 | 0.123 |
|  | 1 | G♂ | 0.910 | 0.394 | 4.254 | 0.108 | 37.449 | **0.004** | 0.043 | 0.847 | 0.001 | 0.975 |
|  | 1 | RO♀ | 4.159 | 0.111 | 2.554 | 0.185 | 15.532 | **0.017** | 1.533 | 0.283 | 0.059 | 0.820 |
|  | 1 | RO♂ | 11.587 | **0.027** | 1.225 | 0.331 | 0.444 | 0.542 | 1.781 | 0.253 | 0.039 | 0.853 |
| *DaGSTe10* | 1 | A♀ | 2.523 | 0.187 | 0.025 | 0.881 | 26.870 | **0.007** | 0.155 | 0.713 | 22.561 | **0.009** |
|  | 1 | A♂ | 0.180 | 0.693 | 0.612 | 0.478 | 0.760 | 0.432 | 1.084 | 0.357 | 0.509 | 0.515 |
|  | 1 | G♀ | 45.863 | **0.002** | 0.594 | 0.484 | 0.080 | 0.791 | 7.035 | 0.057 | 13.891 | **0.020** |
|  | 1 | G♂ | 8.792 | **0.041** | 0.039 | 0.853 | 0.292 | 0.618 | 0.212 | 0.669 | 5.064 | 0.088 |
|  | 1 | RO♀ | 0.047 | 0.840 | 1.192 | 0.336 | 0.618 | 0.476 | 0.326 | 0.599 | 0.454 | 0.537 |
|  | 1 | RO♂ | 81.894 | **0.001** | 0.207 | 0.673 | 2.884 | 0.165 | 7.634 | 0.051 | 0.959 | 0.383 |
| *DaGSTe11* | 1 | A♀ | 0.448 | 0.540 | 3.259 | 0.145 | 13.137 | **0.022** | 3.176 | 0.149 | 0.794 | 0.423 |
|  | 1 | A♂ | 0.045 | 0.843 | 0.442 | 0.543 | 1.141 | 0.346 | 0.711 | 0.447 | 0.913 | 0.393 |
|  | 1 | G♀ | 14.633 | **0.019** | 0.107 | 0.761 | 1.058 | 0.362 | 0.470 | 0.531 | 2.957 | 0.161 |
|  | 1 | G♂ | 0.319 | 0.602 | 3.159 | 0.150 | 5.313 | 0.082 | 0.001 | 0.976 | 0.059 | 0.820 |
|  | 1 | RO♀ | 0.031 | 0.870 | 0.054 | 0.827 | 0.279 | 0.625 | 0.635 | 0.470 | 18.803 | 0.012 |
|  | 1 | RO♂ | 8.114 | **0.046** | 0.036 | 0.859 | 1.565 | 0.279 | 0.791 | 0.424 | 2.334 | 0.201 |
| *DaGSTe12* | 1 | A♀ | 11.547 | **0.027** | 17.272 | **0.014** | 1.607 | 0.274 | 2.591 | 0.183 | 13.234 | **0.022** |
|  | 1 | A♂ | 0.184 | 0.690 | 0.646 | 0.467 | 1.238 | 0.328 | 0.789 | 0.425 | 0.735 | 0.440 |
|  | 1 | G♀ | 23.505 | **0.008** | 0.245 | 0.647 | 0.307 | 0.609 | 18.507 | **0.013** | 4.070 | 0.114 |
|  | 1 | G♂ | 3.708 | 0.126 | 7.289 | 0.054 | 34.166 | **0.004** | 0.114 | 0.753 | 0.033 | 0.864 |
|  | 1 | RO♀ | 2.779 | 0.171 | 0.940 | 0.387 | 107.896 | **<0.001** | 0.452 | 0.538 | 0.679 | 0.456 |
|  | 1 | RO♂ | 14.668 | **0.019** | 1.316 | 0.315 | 1.482 | 0.290 | 0.416 | 0.554 | 0.481 | 0.526 |
| *DaGSTs3* | 1 | A♀ | 1.598 | 0.275 | 1.508 | 0.287 | 3.231 | 0.147 | 1.481 | 0.291 | 0.617 | 0.476 |
|  | 1 | A♂ | 0.767 | 0.431 | 1.311 | 0.316 | 1.508 | 0.287 | 1.849 | 0.246 | 0.521 | 0.510 |
|  | 1 | G♀ | 21.664 | **0.010** | 0.003 | 0.959 | 1.349 | 0.310 | <0.001 | 0.987 | 0.044 | 0.844 |
|  | 1 | G♂ | 4.375 | 0.105 | 1.683 | 0.264 | 177.116 | **<0.001** | 2.071 | 0.224 | 5.371 | 0.081 |
|  | 1 | RO♀ | 12.396 | **0.024** | 2.559 | 0.185 | 59.024 | 0.002 | 3.490 | 0.135 | 0.154 | 0.715 |
|  | 1 | RO♂ | <0.001 | 0.993 | 0.331 | 0.596 | 6.719 | 0.061 | 2.446 | 0.193 | 0.208 | 0.672 |
| *DaGSTs4* | 1 | A♀ | 4.671 | 0.097 | 3.319 | 0.143 | 1.720 | 0.260 | 0.650 | 0.465 | 2.686 | 0.177 |
|  | 1 | A♂ | 0.072 | 0.801 | 0.522 | 0.510 | 1.595 | 0.275 | 1.354 | 0.309 | 0.417 | 0.554 |
|  | 1 | G♀ | 14.362 | **0.019** | 0.632 | 0.471 | 0.151 | 0.717 | 3.130 | 0.152 | 0.836 | 0.412 |
|  | 1 | G♂ | 4.249 | 0.108 | 7.705 | 0.050 | 114.714 | **<0.001** | 0.250 | 0.644 | 2.646 | 0.179 |
|  | 1 | RO♀ | 4.968 | 0.090 | 0.679 | 0.456 | 54.188 | **0.002** | 0.256 | 0.639 | 3.065 | 0.155 |
|  | 1 | RO♂ | 28.022 | **0.006** | 0.283 | 0.623 | 1.511 | 0.286 | <0.001 | 0.989 | 1.755 | 0.256 |
| *DaGSTs6* | 1 | A♀ | 6.154 | 0.068 | 0.074 | 0.799 | 1.900 | 0.240 | 0.037 | 0.856 | 2.083 | 0.222 |
|  | 1 | A♂ | 0.260 | 0.637 | 0.457 | 0.536 | 1.112 | 0.351 | 1.628 | 0.271 | 0.240 | 0.650 |
|  | 1 | G♀ | 0.094 | 0.775 | 1.877 | 0.243 | 2.237 | 0.209 | 0.268 | 0.632 | 0.061 | 0.818 |
|  | 1 | G♂ | 1.703 | 0.262 | 4.505 | 0.101 | 8.173 | **0.046** | 0.805 | 0.420 | 0.204 | 0.675 |
|  | 1 | RO♀ | 0.798 | 0.422 | 2.051 | 0.225 | 60.187 | **0.001** | 1.454 | 0.294 | 0.091 | 0.778 |
|  | 1 | RO♂ | 0.610 | 0.478 | 1.467 | 0.293 | 1.269 | 0.323 | 7.971 | **0.048** | 0.001 | 0.983 |
| *DaGSTs7* | 1 | A♀ | 0.049 | 0.836 | 5.540 | 0.078 | 3.590 | 0.131 | 3.830 | 0.122 | 1.075 | 0.358 |
|  | 1 | A♂ | 0.970 | 0.381 | 1.114 | 0.351 | 1.086 | 0.356 | 1.551 | 0.281 | 0.857 | 0.407 |
|  | 1 | G♀ | 1.989 | 0.231 | 3.587 | 0.131 | 2.726 | 0.174 | 0.024 | 0.885 | 0.167 | 0.704 |
|  | 1 | G♂ | 3.104 | 0.153 | 8.685 | **0.042** | 90.261 | **0.001** | 1.352 | 0.310 | 4.641 | 0.098 |
|  | 1 | RO♀ | 1.918 | 0.238 | 0.315 | 0.605 | 3.348 | 0.141 | 2.768 | 0.172 | 0.197 | 0.680 |
|  | 1 | RO♂ | 0.002 | 0.970 | 0.085 | 0.785 | 2.947 | 0.161 | 3.464 | 0.136 | 0.126 | 0.740 |
| *DaGSTt2* | 1 | A♀ | 5.342 | 0.082 | 1.983 | 0.232 | 1.336 | 0.312 | 2.766 | 0.172 | 2.853 | 0.166 |
|  | 1 | A♂ | 0.096 | 0.772 | 0.534 | 0.505 | 1.166 | 0.341 | 1.406 | 0.301 | 0.276 | 0.627 |
|  | 1 | G♀ | 34.424 | **0.004** | 0.653 | 0.464 | 1.092 | 0.355 | 1.076 | 0.358 | 69.785 | **0.001** |
|  | 1 | G♂ | 0.355 | 0.583 | 4.894 | 0.091 | 41.584 | **0.003** | 0.062 | 0.816 | 5.004 | 0.089 |
|  | 1 | RO♀ | 3.564 | 0.132 | 2.436 | 0.194 | 22.533 | **0.009** | 1.801 | 0.251 | 0.102 | 0.766 |
|  | 1 | RO♂ | 27.675 | **0.006** | 0.842 | 0.411 | 2.671 | 0.178 | 0.099 | 0.769 | 4.241 | 0.109 |
| *DaGSTt3* | 1 | A♀ | 0.912 | 0.394 | 1.344 | 0.311 | 4.769 | 0.094 | 0.847 | 0.409 | 0.369 | 0.576 |
|  | 1 | A♂ | 0.543 | 0.502 | 0.992 | 0.376 | 0.523 | 0.510 | 1.801 | 0.251 | 0.164 | 0.706 |
|  | 1 | G♀ | 0.261 | 0.637 | 0.429 | 0.548 | 0.609 | 0.479 | 0.628 | 0.472 | 0.059 | 0.820 |
|  | 1 | G♂ | 0.018 | 0.899 | 0.896 | 0.397 | 7.972 | **0.048** | 0.329 | 0.597 | 0.884 | 0.400 |
|  | 1 | RO♀ | 9.667 | **0.036** | 3.471 | 0.136 | 29.604 | **0.006** | 9.838 | **0.035** | 0.054 | 0.828 |
|  | 1 | RO♂ | 0.686 | 0.454 | 0.115 | 0.752 | 2.882 | 0.165 | 2.357 | 0.200 | <0.001 | 0.998 |

Note: A♀: Female Antenna; A♂: Male Antenna; G♀: Female Gut; G♂: Male Gut; RO♀: Female Reproductive Organ; RO♂: Male Reproductive Organ. *P* values in bold indicate significant effects.
